# Inhibition of *Arabidopsis thaliana* CIN‐like TCP transcription factors by *Agrobacterium* T‐DNA‐encoded 6B proteins

**DOI:** 10.1111/tpj.14591

**Published:** 2019-12-05

**Authors:** Thomas Potuschak, Javier Palatnik, Carla Schommer, Nicolas Sierro, Nikolai V. Ivanov, Yerim Kwon, Pascal Genschik, Jean‐Michel Davière, Léon Otten

**Affiliations:** ^1^ Institut de Biologie Moléculaire des Plantes (IBMP) Rue du Général Zimmer 12 67084 Strasbourg France; ^2^ IBR‐CONICET Predio CCT Ocampo y Esmeralda s/n 2000 Rosario Argentina; ^3^ PMI R&D Philip Morris Products S. A. Quai Jeanrenaud 5 2000 Neuchâtel Switzerland

**Keywords:** *Nicotiana otophora*, natural transformant, *6b* oncogene, *jaw‐D* phenotype, *TCP* genes

## Abstract

*Agrobacterium* T‐DNA‐encoded 6B proteins cause remarkable growth effects in plants. *Nicotiana otophora* carries two cellular T‐DNAs with three slightly divergent *6b* genes (*TE‐1‐6b‐L*, *TE‐1‐6b‐R* and *TE‐2‐6b*) originating from a natural transformation event. In *Arabidopsis thaliana*, expression of 2×*35S:TE‐2‐6b*, but not 2×*35S:TE‐1‐6b‐L* or 2×*35S:TE‐1‐6b‐R*, led to plants with crinkly leaves, which strongly resembled mutants of the miR319a/*TCP* module. This module is composed of *MIR319A* and five *CIN*‐like *TCP* (*TEOSINTHE BRANCHED1, CYCLOIDEA* and *PROLIFERATING CELL NUCLEAR ANTIGEN BINDING FACTOR*) genes (*TCP2*, *TCP3*, *TCP4*, *TCP10* and *TCP24*) targeted by miR319a. The *CIN*‐like *TCP* genes encode transcription factors and are required for cell division arrest at leaf margins during development. *MIR319A* overexpression causes excessive growth and crinkly leaves. *TE‐2‐6b* plants did not show increased miR319a levels, but the mRNA levels of the TCP4 target gene *LOX2* were decreased, as in *jaw‐D* plants. Co‐expression of green fluorescent protein (GFP)‐tagged TCPs with native or red fluorescent protein (RFP)‐tagged TE‐6B proteins led to an increase in TCP protein levels and formation of numerous cytoplasmic dots containing 6B and TCP proteins. Yeast double‐hybrid experiments confirmed 6B/TCP binding and showed that TE‐1‐6B‐L and TE‐1‐6B‐R bind a smaller set of TCP proteins than TE‐2‐6B. A single nucleotide mutation in TE‐1‐6B‐R enlarged its TCP‐binding repertoire to that of TE‐2‐6B and caused a crinkly phenotype in Arabidopsis. Deletion analysis showed that TE‐2‐6B targets the TCP4 DNA‐binding domain and directly interferes with transcriptional activation. Taken together, these results provide detailed insights into the mechanism of action of the *N. otophora* TE‐encoded *6b* genes.

## Significance Statement

It was found previously that the *Agrobacterium*‐derived *TE‐2‐6b* gene from *Nicotiana otophora* induces strong growth effects in *Nicotiana tabacum*. However, the molecular mechanism has remained unknown. We show here that *TE‐2‐6b* induces a *jaw*‐*D*‐like phenotype in Arabidopsis – known to result from a decrease in CIN‐like TCPs – and that different TE‐6B proteins interact with different TCP subsets, thus providing insights into the molecular mechanism by which TE‐6B proteins stimulate plant growth.

## Introduction

Pathogenic *Agrobacterium* strains manipulate plant growth and metabolism by transferring specific DNA fragments (transferred DNAs or T‐DNAs) to the nuclei of infected plant cells (Zhu *et al.*, 2000; Gelvin, 2017; Barton *et al.*, 2018). These T‐DNAs are located on large tumor‐inducing plasmids or root‐inducing plasmids and are highly diverse in sequence and structure. Some T‐DNA genes cause undifferentiated growth, leading to crown gall tumors (as in the case of *Agrobacterium tumefaciens* and *Agrobacterium vitis*), while others induce abnormal roots (as for *Agrobacterium rhizogenes*), which can regenerate into fertile plants (White *et al.*, 1983; Chen and Otten, 2017; Matveeva and Otten, 2019). Additional T‐DNA genes encode enzymes for the synthesis of unusual molecules called opines, which are used by agrobacteria as nutrients. Among the growth‐inducing T‐DNA genes, *iaaM* and *iaaH* encode enzymes that catalyze the synthesis of indole‐3‐acetic acid (an auxin), whereas *ipt* encodes an enzyme involved in the synthesis of isopentenyl adenine (a cytokinin). Together, the *iaa* and *ipt* genes induce tumors (Zhu *et al.*, 2000). In addition to *iaa* and *ipt*, a large family of highly divergent T‐DNA genes, called *plast* genes (for phenotypic plasticity), can also lead to growth induction or modification, as shown by expression in model plants, such as *Nicotiana tabacum* and *Arabidopsis thaliana* (Levesque *et al.*, 1988; Britton *et al.*, 2008; Otten, 2018). The *A. tumefaciens A‐6b plast* oncogene induces tumors in a limited group of plant species (Hooykaas *et al.*, 1988). Additional *6b* genes include *C‐6b* and *AK‐6b* in *A. tumefaciens* and *T‐6b*, *S‐6b*, *CG*‐*6b* and *AB*‐*6b* in *A. vitis* (Helfer *et al.*, 2002; Otten, 2018). *AK‐6b* induces a serrated phenotype in Arabidopsis (Terakura *et al.*, 2006). In *N. tabacum*, *AB‐6b* and *T‐6b* induce a complex set of growth changes, collectively called the enation syndrome (Helfer *et al.*, 2003). This enation syndrome refers to the presence of double leaves (enations) and double flowers (catacorollas), tubular leaves, ectopic vascular bundles and ectopic leaf primordia growing from the base of large glandular trichomes (Helfer *et al.*, 2003; Grémillon *et al.*, 2004; Chen and Otten, 2016). The *T‐6b* gene also causes root swelling and localized sucrose uptake (Grémillon *et al.*, 2004; Clément *et al.*, 2007), accompanied by rapid changes in the division pattern of the root apical meristem (Pasternak *et al.*, 2017).

Yeast double‐hybrid studies have identified three *N. tabacum* 6B‐interacting proteins (NtSIPs): transcription factor‐like NtSIP1 (Kitakura *et al.*, 2002), histone 3 (previously called NtSIP3; Terakura *et al.*, 2007) and NtSIP2, which resembles the TNP1 protein of a transposable element (Kitakura *et al.*, 2008). It has been proposed that binding of 6B proteins to these nuclear proteins interferes with transcription, thereby leading to *6b* phenotypes. AK‐6B and AB‐6B have been crystallized and were reported by Wang *et al.* (2011) to have ADP ribosylation activity. The same authors showed that this activity targeted the RNA‐silencing factors AGO1 and SERRATE. In addition, *AK‐6b *Arabidopsis plants showed decreased levels of miR162, miR164, miR165/166 and miR319a and increased levels of the corresponding target transcripts (miR164, *DCL1*; miR165/166, *CUC1*; miR319a, *NAC1*, *REV* and *TCP4*). It was proposed that these changes caused the *AK‐6b* serrated phenotype (Wang *et al.*, 2011). For reviews on the mechanism of action of 6B proteins see Ishibashi *et al.* (2014), Ito and Machida (2015) and Otten (2018).

Recently, three *6b* genes were identified in a naturally transgenic plant species, *Nicotiana otophora* (Chen *et al.*, 2018). The original cellular T‐DNA (cT‐DNA) was inserted as a single T‐DNA fragment (called TE) consisting of a partial inverted repeat, with each repeat containing an intact *6b* gene. Later duplication yielded TE‐1 and TE‐2. The left‐ and right‐hand repeats of TE‐1 carry *TE‐1‐6b‐L* and *TE‐1‐6b‐R*, respectively. TE‐2 underwent a deletion of its right arm and carries *TE‐2‐6b* on the remaining left part. TE‐1‐6B‐L, TE‐1‐6B‐R and TE‐2‐6B are only slightly diverged but quite different from the other 6B proteins (54% identity to the closest homolog, T‐6B; Chen *et al.*, 2018). The activities of the TE *6b* genes were studied by expression in the closely related species *N. tabacum* – which lacks the TE regions – by using the constitutive 2×35S promoter. The *TE‐6b* phenotypes strongly differed from the earlier observed *6b* phenotype associated with the enation syndrome (Chen *et al.*, 2018). After this study, it was noted that the *TE‐1‐6b‐L* and *TE‐2‐6b* plants had been inverted. This has been corrected in an erratum note (Chen *et al*., 2018). *TE‐1‐6b‐L* and *TE‐1‐6b‐R* plants showed reinforced minor leaf veins and modified petiole wings (‘weak phenotype’), whereas *TE‐2‐6b* plants also showed outgrowth of leaf margins, flower modifications, abundant trichome development on leaves and vivipary (‘strong phenotype’). No effects were seen at the root level.

These *N. tabacum TE‐6b* phenotypes did not resemble any *N. tabacum* mutant phenotype, and therefore yielded no obvious clues about the molecular mechanism by which *TE‐6b* genes modify plant growth. Because of the remarkable outgrowth of the leaf margins, we speculated (Chen *et al.*, 2018) that this phenotype could be similar to the *jaw‐D* phenotype in *A. thaliana*. In *jaw‐D* mutants, the *MIR319A* gene is ectopically activated by an enhancer cassette promoter (Palatnik *et al.*, 2003). In wild‐type Arabidopsis plants, miR319a controls the transcript levels of five *CIN*‐like class II *TCP* genes (*TCP2*, *TCP3*, *TCP4*, *TCP10* and *TCP24*). Three additional *CIN*‐like *TCP* genes (*TCP5*, *TCP13* and *TCP17*) have similar functions but are not affected by miR319a. Altogether, Arabidopsis contains 24 *TCP* genes, which encode transcription factors regulating various target genes (Martín‐Trillo and Cubas, 2009; Li, 2015; Sarvepalli and Nath, 2018). Mutations in *CIN*‐like *TCP* genes, their downregulation by artificial miRNAs and the use of a chimeric TCP repressor in various species all lead to crinkly phenotypes (Koyama *et al.*, 2007; Efroni *et al.*, 2008; Schommer *et al.*, 2008; Koyama *et al.*, 2010; Alvarez *et al.*, 2016; Bresso *et al.*, 2018). The *CIN*‐like *TCP* genes are partially redundant, as single mutants have only weak phenotypes, whereas multiple mutants show increasingly crinkly leaves (Schommer *et al.*, 2008; Koyama *et al.*, 2010; Bresso *et al.*, 2018). It has been proposed that the *CIN*‐like *TCP* genes control cell division arrest at the leaf margins in the early stages of leaf development and thereby ensure the flatness of the leaf (Nath *et al.*, 2003; Alvarez *et al.*, 2016). In order to produce its growth effects, the miR319a/*TCP* module might act through activation of the miR164/*CUC* (*CUP‐SHAPED COTYLEDONS*) module (Koyama *et al.*, 2010); however, the precise mechanism remains unknown. Interestingly, the SAP11_AYWB_ (SECRETED AY‐WB PROTEIN 11) effector of Aster Yellows phytoplasma induces witches' broom by binding and destabilizing the *CIN* class II TCP proteins. In Arabidopsis, SAP11_AYWB_ expression under 35S promoter control leads to crinkly leaves and siliques, similar to the phenotype in *jaw‐D* mutants (Sugio *et al.*, 2011, 2014). *Nicotiana benthamiana* plants expressing *SAP11_AYWB_* have wrinkled leaves (Tan *et al.*, 2016) which resemble those of *TE‐2‐6b N. tabacum* plants (Chen *et al.*, 2018). Although SAP11_AYWB_ localizes to the nucleus (Bai *et al.*, 2009; Sugio *et al.*, 2014), other SAP11‐like proteins are evenly distributed throughout the cell (Chang *et al.*, 2018). Given the strong resemblance between the *TE‐2‐6b*, *jaw‐D*/*TCP* and *SAP11* phenotypes, we hypothesized that *TE‐2‐6b* interferes with the miR319a/*TCP* module. We therefore introduced the three *TE‐6b* genes in *A. thaliana* ecotype Col‐0 and investigated the miR319a/*TCP* hypothesis in detail.

## Results

### Phenotypes of Arabidopsis Col‐0 plants transformed with the *TE‐6b* genes

**Figure 1:**
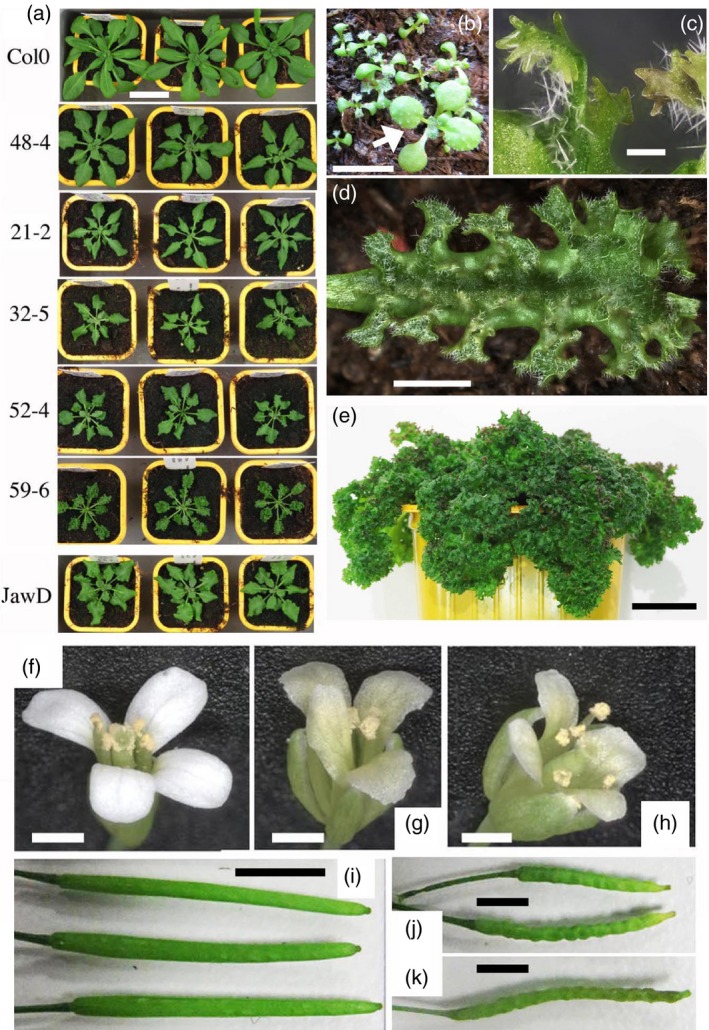
Col‐0 *TE‐2‐6b* and Col‐0 *jaw‐2D* phenotypes. (a) Col‐0, Col‐0 *TE‐2‐6b* and Col‐0 *jaw‐2D* transformants. Five different homozygous *TE‐2‐6b* single‐locus lines 4 weeks after germination, in order of increasingly modified phenotype (top to bottom). Phenotypes vary from weakly crinkled (48‐4) to small and highly crinkled (59‐6). Col‐0 and *jaw‐2D* plants are shown for comparison. (b) Col‐0 *TE‐2‐6b* seedlings 1 week after germination compared with a Col‐0 wild‐type seedling (arrow): *TE‐2‐6b* plants are smaller, with epinastic cotyledons and epinastic and crinkly first leaves. (c) Finger‐like outgrowths along the leaf edges of a *TE‐2‐6b* 59‐6 plant (fifth phytomer), 8 weeks after germination. (d) Leaf of a *TE‐2‐6b* 59‐6 plant (fifth phytomer), 4 weeks after germination. Note the fractal folds‐on‐folds structure. (e) Highly crinkled leaves of a *TE‐2‐6b* 59‐6 plant 8 weeks after germination. (f) Col‐0 flower. (g) Col‐0 *TE‐2‐6b* flower: note the loss of white pigmentation and irregular petal edges. (h) *jaw‐2D* flower. (i) Col‐0 siliques. (j) Col‐0 *TE‐2‐6b* siliques: note the crinkling. (k) *jaw‐2D* silique. Scale bars: 3 cm (a), 1 cm (b,d), 0.2 mm (c), 1.5 cm (e), 2 mm (f,g,h), 0.4 cm (i,j,k).

The effects of the ‘weak *TE‐6b* genes’ *TE‐1‐6b‐L* and *TE‐1‐6b‐R* and the ‘strong *TE‐6b* gene’ *TE‐2‐6b* were tested in the model plant *A. thaliana* by stable transformation. Primary *TE‐1‐6b‐L* (40 plants) and *TE‐1‐6b‐R* (72 plants) transformants did not show any obvious phenotype; however, 20 out of 23 *TE‐2‐6b* transformants had crinkly leaves. Five independent, homozygous, single‐locus *TE‐2‐6b* lines were obtained: 21‐2, 32‐5, 48‐4, 52‐4 and 59‐6 (Figure 1a). They varied from relatively normal plants (48‐4) to plants with extremely crinkly leaves (59‐6). At early stages, *TE‐2‐6b* plantlets were smaller than Col‐0 plants and had epinastic cotyledons and primary leaves (Figure 1b). At later stages, the leaf edges of *TE‐2‐6b* plants formed numerous, very small finger‐like outgrowths (Figure 1c) and showed a fractal pattern of folds‐on‐folds (Figure 1d). At even later stages, the leaves showed extreme folding and remained green (Figure 1e), as in *TE‐2‐6b N. tabacum* (Chen *et al.*, 2018). Col‐0 *TE‐2‐6b* petals were off‐white and formed irregular margins (Figure 1f,g). A similar change in petal color was found in *TE‐2‐6b N. tabacum* (Chen *et al.*, 2018). Col‐0 *TE‐2‐6b* siliques were crinkly, with bulges at the position of the seeds (Figure 1i,j). The similarity of effects of the *TE‐2‐6b* gene in *A. thaliana* and *N. tabacum* indicated that the TE‐2‐6B protein targets a conserved plant growth mechanism.

Col‐0 *TE‐2‐6b* plants differed from Col‐0 *AB‐6b* plants, which showed tubular structures growing out from the abaxial leaf surface (Helfer *et al.*, 2003). Young Col‐0 *TE‐2‐6b* plants somewhat resembled *AK‐6b* plants, the phenotype of which has been described as ‘serrate’ (Terakura *et al.*, 2006). However, at later stages, Col‐0 *TE‐2‐6b* plants no longer resembled serrate mutants, as their leaf margins continued to grow. Instead, they strikingly resembled Arabidopsis Col‐0 *jaw‐D* mutants (Weigel *et al.*, 2000; Palatnik *et al.*, 2003). Figure 1 shows *jaw‐2D* plants (Figure 1a), a *jaw‐2D* flower (Figure 1h) and a *jaw‐2D* silique (Figure 1k) for comparison. As the Arabidopsis* jaw‐D* phenotype is quite remarkable and unique and has so far only been encountered in plants with diminished CIN‐TCP activity, our findings reinforced the hypothesis that the *TE‐2‐6b* gene targets the miR319a/*TCP* module. We first tested whether *TE‐2‐6b* expression in Col‐0 led to an increase in miR319a levels, as in the case of the *jaw*‐*D* mutants.

### Steady‐state levels of miR319a in Col‐0 *TE‐2‐6b* plants are not increased

**Figure 2:**
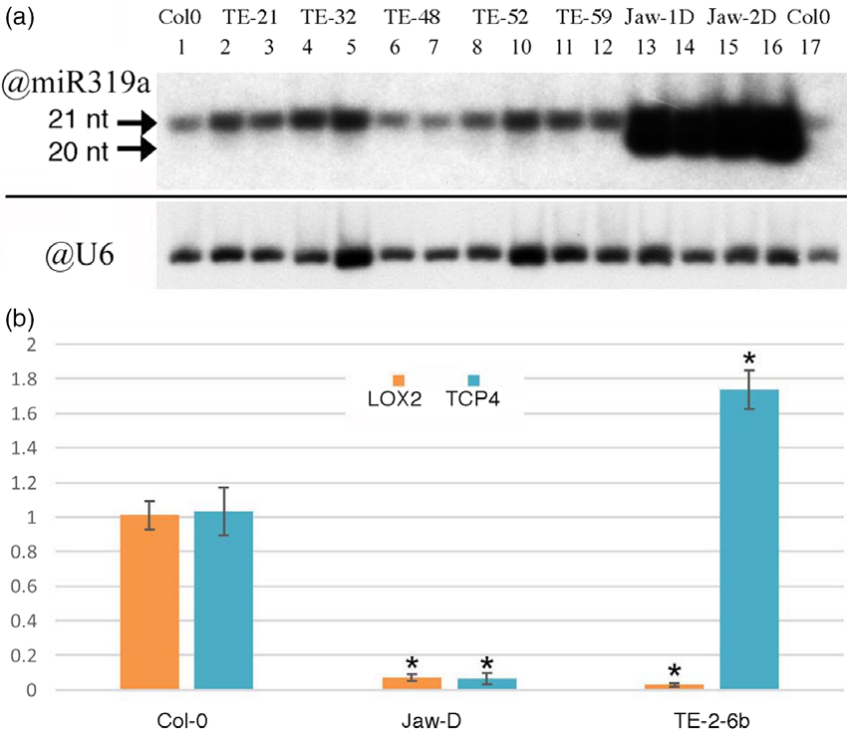
miR319a levels and transcript levels of *TCP4* and *LOX2* in the Col‐0, *jaw‐D* and *TE‐2‐6b* lines. (a) miR319a levels of Col‐0, *TE‐2‐6b* lines 21‐2, 32‐5, 48‐4, 52‐4 and 59‐6, and *jaw‐1D* and *jaw‐2D* mutants. Two plants were analyzed for each line. The 21‐nucleotide (nt) band corresponds to the cross‐reacting miR159 (Palatnik *et al*., 2007) and the 20‐nt band corresponds to miR319a. Only *jaw‐1D* and *jaw‐2D* plants show detectable levels of miR319a. U6 was used as a loading control. (b) Relative expression levels of the TCP4 target gene *LOX2* in Col‐0, *jaw‐2D* and *TE‐2‐6b* line 21‐2. *TCP4* expression is significantly lower in *jaw‐2D* than in Col‐0, as is the expression of the TCP4 target gene *LOX2*. In *TE‐2‐6b* line 21‐2, *TCP4* expression is higher than in Col‐0, but *LOX2* expression is very low. Ordinate numbers are fold‐change. Unpaired Student's *t*‐test was used to determine significant differences relative to Col‐0 values, indicated by an asterisk. *P* < 0.05.

Steady‐state levels of miR319a were investigated in the Col‐0 *TE‐2‐6b* homozygous single‐locus lines 21‐2, 32‐5, 48‐4, 52‐4 and 59‐6 (Figure 1a). Col‐0 plants were used as wild‐type controls and *jaw‐1D* and *jaw‐2D* as overexpression controls. The results are shown in Figure 2(a). The 20‐nucleotide (nt) band corresponds to miR319a; the 21‐nt band is a result of the cross‐reacting miR159 (Palatnik *et al.*, 2007). Whereas *jaw‐1D* and *jaw‐2D* showed an expected strong increase in miR319a relative to Col‐0 plants (Palatnik *et al.*, 2003), the miR319a levels in the different *TE‐2‐6b* lines remained below detection levels (as in the Col‐0 control) despite the fact that four of the five *TE‐2‐6b* lines had a stronger phenotype than the *jaw‐D* lines. We next tested *TCP4* expression levels and the expression of the TCP4 target gene *LOX2*.

### Expression of the TCP4 target gene *LOX2* in *TE‐2‐6b* plants is decreased

In *jaw‐D* mutants, the steady‐state mRNA levels of the miR319a target gene TCP4 and the downstream TCP4 target gene *LOX2* are very low (Schommer *et al.*, 2008). In the present study, *TCP4* and *LOX2* expression were measured in *TE‐2‐6b* line 21‐2. The *TCP4* expression level in *TE‐2‐6b* line 21‐2 was not decreased (as in the *jaw‐2D* mutant) but actually higher than that in Col‐0 (Figure 2b). However, in spite of this, the *LOX2* mRNA levels were very low, as in the *jaw‐2D* mutant. Thus, the TCP4 target gene *LOX2* is not activated despite the relatively high *TCP4* mRNA levels. A more complete view of the transcription patterns of *TE‐2‐6b* and *jaw‐2D* plants was obtained by RNA sequencing.

### Comparison between the transcriptomes of Col‐0, *jaw‐2D* and different Col‐0 *TE‐2‐6b* lines

**Figure 3:**
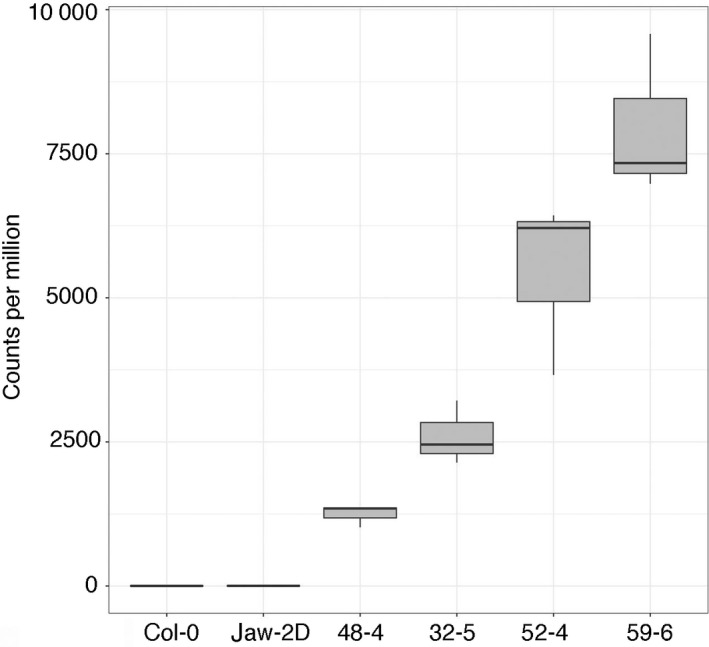
Expression of the *TE‐2‐6b* gene in Col‐0, *jaw‐2D* and Col‐0 *TE‐2‐6b* lines 48‐4, 32‐5, 52‐4 and 59‐6. Expression is measured as normalized counts, and the Col‐0 *TE‐2‐6b* lines are ordered by the severity of their crinkly phenotype. Two replicates were used for Col‐0, and three replicates for each of the other lines.

**Figure 4:**
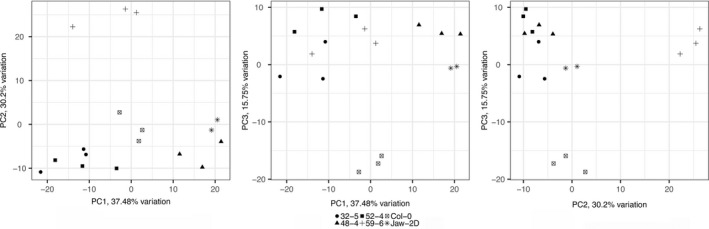
Principal component analysis of the transcriptomes of Col‐0, *jaw‐2D* and the Col‐0 *TE‐2‐6b* lines 48‐4, 32‐5, 52‐4 and 59‐6. Scatter plots show the contributions of the first and second (left), first and third (center) and second and third (right) components. The first principal component separates the Col‐0 *TE‐2‐6b* line and *jaw‐2D* from the others, and the second and third principal components separate the Col‐0 *TE‐2‐6b* line 59‐6 and Col‐0 from the other lines. Percentages on the *x* and *y* axes indicate the contribution of each component in explaining the variation in the samples. Col‐0 samples were in duplicate and the other samples were in triplicate.

The transcriptomes of Col‐0, *jaw‐2D* and Col‐0 *TE‐2‐6b* lines 48‐4, 32‐5, 52‐4 and 59‐6 were sequenced (see Experimental Procedures). First of all, the relative levels of *TE‐2‐6b* reads in the different lines correlated well with the severity of the crinkly phenotype (Figure 3). Second, principal component analysis showed that the transcriptome of *jaw‐2D* most closely resembled that of line 48‐4, as the first principal component separated these two lines from the others (Figure 4). This finding correlates well with the phenotypic similarity between *jaw‐2D* and line 48‐4 (Figure 1a). The second and third principal component separated line 59‐6 and Col‐0 from the other lines. We next compared the *jaw‐2D* line with the phenotypically most similar *TE‐2‐6b* line, 48‐4. Pearson correlation coefficients between the expression of all genes and *TE‐2‐6b* across all samples were calculated to identify genes that are potentially co‐regulated with *TE‐2‐6b*. Figure S1 in the online Supporting Information shows the difference in gene expression (relative to the mean Col‐0 gene expression) for the 50 most and 50 least correlated genes in the Col‐0, *jaw‐2D* and line 48‐4 samples. Figure S2 shows the difference in gene expression (relative to the mean Col‐0 gene expression) for the 100 genes whose expression varies the most across the Col‐0, *jaw‐2D* and line 48‐4 samples. Four main groups of genes can be identified. The first set of genes (marked 1) shows variable or more expression in *jaw‐2D* and 48‐4. The second group (marked 2) is less expressed in *jaw‐2D* but not in line 48‐4. This group includes *TCP2*, *TCP3* and *TCP4* (marked by arrows) and a few non‐*TCP* genes. Those known to be regulated by *CIN‐TCP* genes (see below) are marked by dots (*LURP1*, *PCC* and *CML10*). A third set of genes (marked 3) is less expressed in both *jaw‐2D* and line 48‐4. The fourth group (marked 4) is less expressed in line 48‐4, but not in *jaw‐2D* and contains *LHCB1.1*, *LHCB2.1* and *LHCB2.4*.

While the first and third sets of genes might be linked to the crinkly phenotype itself, and therefore see their expression modified in a similar manner in both *jaw‐2D* and line 48‐4, the second and last sets of genes are more likely linked to the mechanism by which *jaw‐2D* and *TE‐2‐6b* control this phenotype.

Earlier studies have identified a number of CIN‐TCP target genes (Schommer *et al.*, 2008; Sarvepalli and Nath, 2018). If the *TE‐2‐6b* gene interferes with CIN‐TCP function it can be expected that *TE‐2‐6b* expression affects the transcript levels of these CIN‐TCP targets. A heat map for the transcripts of the *CIN‐TCP* genes and CIN‐TCP target genes for Col‐0, *jaw‐2D*, 48‐4, 32‐5, 52‐4 and 59‐6 is shown in Figure S3. Most CIN‐TCP target genes behave similarly in *jaw‐2D* and 48‐4. However, several genes are downregulated in *jaw‐2D* but unchanged or slightly upregulated in 48‐4. This group contains not only *TCP2*, *TCP3*, *TCP4*, *TCP10* and *TCP24* (marked with arrows) but also some CIN‐TCP target genes. These CIN‐TCP target gene transcripts are decreased in one or more of the other *TE‐2‐6b* lines (32‐5, 52‐4 and 59‐6) but *ACX1* (acyl‐coenzyme A oxidase, marked by an asterisk), *TCP2*, *TCP3*, *TCP4*, *TCP10* and *TCP24* remain unchanged or are slightly upregulated in all *TE‐2‐6b* lines. Thus, *TE‐2‐6b* expression does not affect *CIN‐TCP* gene expression but modifies the expression of most of the CIN‐TCP target genes. The lack of change in *ACX1* expression in the *TE‐2‐6b* lines remains unexplained.

Since *TE‐2‐6b* expression in Arabidopsis does not act on miR319a levels or diminish the transcript levels of the miR319a targets *TCP2*, *TCP3*, *TCP4*, *TCP10* and *TCP24* but leads to a decrease of most of the CIN‐TCP target gene transcripts, we hypothesized that *TE‐2‐6b* expression affects the function of the CIN‐TCP proteins as in the case of the SAP11‐like proteins (Sugio *et al.*, 2011). In a first step, we studied the effect of *TE‐2‐6b* on TCP4, through *TE‐6b*/*TCP* co‐expression experiments in *N. benthamiana*.

### Co‐expression of *TE‐2‐6b* and *TCP4* in *N. benthamiana* causes an increase in TCP4 levels

**Figure 5:**
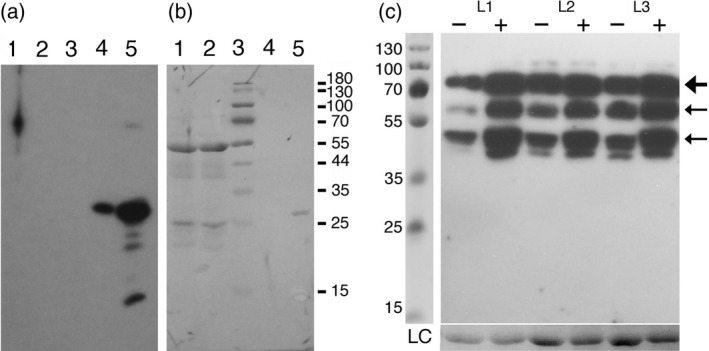
TCP4:GFP protein levels in infiltrated *Nicotiana benthamiana* leaves, in the absence and presence of TE‐2‐6B. (a), (b) Control experiment. (a) GFP detection with polyclonal anti‐GFP antibodies. (b) Coomassie staining. Lanes 1 and 2: extracts of non‐infiltrated *N. benthamiana* leaves. These do not react with the anti‐GFP antibodies. Lane 3: molecular weight marker. Lanes 4 and 5: purified 6× HIS‐GFP‐BD‐CVIM produced in *Escherichia coli* (10 and 50 ng, respectively). (c) Extracts of *N. benthamiana* leaves infiltrated with different constructs. Three successive leaves (L1, L2 and L3, numbered from old to young) of the same plant were tested. Lanes marked −: co‐infiltration with *35S:TCP4:GFP* and empty vector strain LBA4404(pBI121.2); lanes marked +: co‐infiltration with *35S:TCP4:GFP* and 2×*35S:TE‐2‐6b*. In the absence of 2×*35S:TE‐2‐6b*, the leaf extracts show a band with the size expected for intact TCP4:GFP (73 kDa; thick arrow) and two major breakdown products (thin arrows). In the presence of 2×*35S:TE‐2‐6b*, the levels of intact TCP4:GFP proteins and their breakdown products are increased. LC, Rubisco large‐subunit loading control.

In this study, the transcript levels of *TCP2*, *TCP3*, *TCP4*, *TCP10* and *TCP24* in *TE‐2‐6b* plants did not diminish, but the transcripts levels of several of their target genes did. This suggests that *TE‐2‐6b* may affect the levels of TCP protein. It is known that the SAP11 effector decreases the levels of various TCP proteins (Sugio *et al.*, 2011). In order to detect the possible effects of *TE‐6b* expression on TCP protein levels, we first investigated TCP4, which is considered to be the most important member of the CIN‐like TCPs (Nag *et al.*, 2009). A *35S:TCP4:GFP* (GFP, green fluorescent protein) gene construct (Palatnik *et al.*, 2007) was expressed in *N. benthamiana*, with or without 2×*35S:TE‐2‐6b* (see Experimental Procedures). The levels of TCP4:GFP were measured by Western blot analysis using anti‐GFP antibodies (Figure 5a–c). The control experiments showed that *N. benthamiana* extracts do not react with the anti‐GFP antibodies, and that these antibodies react with purified 6×HIS‐GFP‐BD‐CVIM protein (Gerber, 2005) (Figure 5a,b). Co‐infiltration experiments with *35S:TCP4:GFP* and an empty vector construct showed the presence of several bands (Figure 5c, lanes marked −), with the largest one corresponding to the predicted size of TCP4:GFP (73 kDa). The smaller bands are most probably degradation products. In co‐infiltration experiments with *35S:TCP4:GFP* and 2×*35S:TE‐2‐6b* the levels of intact TCP4:GFP and its degradation products were increased (Figure 5c, lanes marked +). This increase was unexpected, as both *jaw‐D* and *SAP11* phenotypes are associated with decreased TCP4 levels (Palatnik *et al.*, 2003; Sugio *et al.*, 2011). However, *TE‐2‐6b* expression may influence TCP factors in other ways, for example by binding or by changing their localization. This possibility was tested in the next step.

### Intracellular localization of TCP2:GFP and TCP4:GFP is modified by co‐expression with TE‐6B proteins

**Figure 6:**
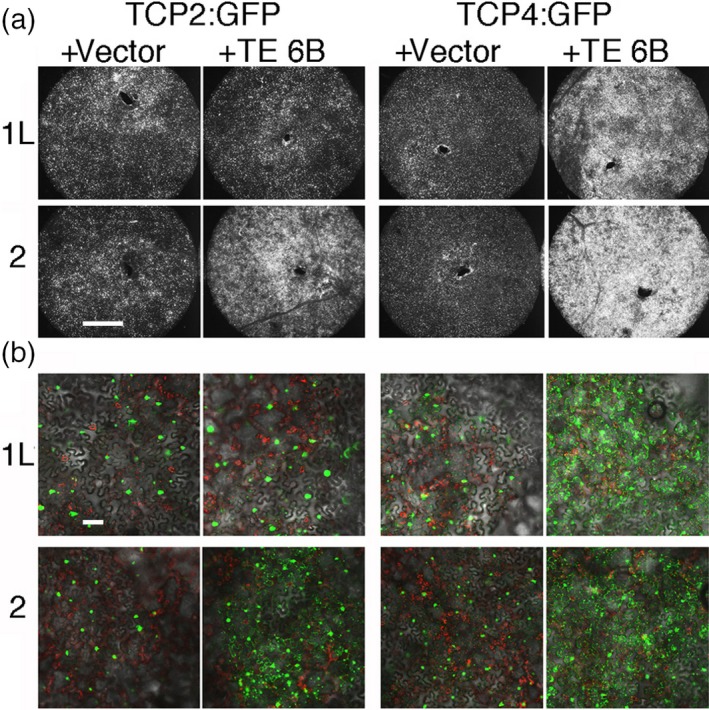
Increase in TCP2:GFP and TCP4:GFP signals and change in intracellular relocalization upon co‐infiltration with 2×*35S:TE‐1‐6b‐L* and 2×*35S:TE‐2‐6b*. *Nicotiana benthamiana* plants were infiltrated with *35S:TCP2:GFP* or *35S:TCP4:GFP* constructs, with either the empty vector strain or one of the *TE‐6b* genes. (a) GFP fluorescence of leaf disks at low magnification. (b) Leaf disks at higher magnification, by confocal analysis. Whereas both 2×*35S:TE‐1‐6b‐L* and 2×*35S:TE‐2‐6b* cause an increase in TCP4:GFP protein levels and relocalization to the cytoplasm, only 2×*35S:TE‐2‐6b* causes such effects on TCP2:GFP. Green, GFP; red, chloroplasts. Scale bars: 1 mm (a), 100 μm (b).

Expression of *35S:TCP2:GFP* and *35S:TCP4:GFP* in *N. benthamiana* leaves in the presence of the empty vector construct (Figure 6a,b) showed their expected nuclear localization (Martín‐Trillo and Cubas, 2009). Co‐infiltration of *35S:TCP4:GFP* along with the ‘weak’ 2×*35S:TE‐1‐6b‐L* or ‘strong’ 2×*35S:TE‐2‐6b* gene caused a significant increase in fluorescence at both low (Figure 6a) and high resolution (Figure 6b), confirming the increase in TCP4:GFP levels seen in the Western blot findings (Figure 5c). Similar effects were seen for the *35S:TCP2:GFP* and 2×*35S:TE‐2‐6b* combination, but not for the *35S:TCP2:GFP* and 2×*35S:TE‐1‐6b‐L* combination. Confocal analysis showed that co‐infiltration of *35S:TCP4:GFP* with 2×*35S:TE‐1‐6b‐L* or 2×*35S:TE‐2‐6b*, and of *35S:TCP2:GFP* with 2×*35S:TE‐2‐6b*, resulted in the formation of multiple cytoplasmic dots (Figure 6b). This suggested that both the ‘weak’ and ‘strong’ TE‐6B proteins retain part of the TCP4 protein in the cytoplasm, possibly by binding to them, and that the TE‐6B proteins may differ in TCP‐binding specificity. TE‐6B/TCP binding would result in co‐localization. This possibility was tested with red fluorescent protein (RFP)‐TE‐6B derivatives.

### The RFP‐tagged TE‐6B derivatives co‐localize with GFP‐tagged TCP proteins

**Figure 7:**
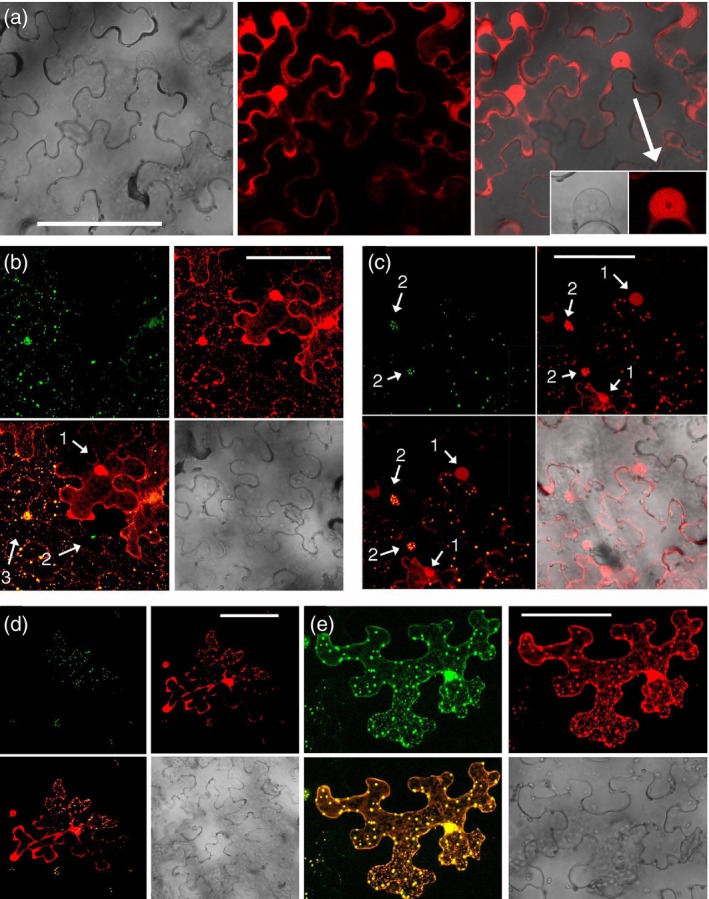
Modification of the localization of GFP‐tagged TCP4 by TE‐2‐6B:RFP in *Nicotiana benthamiana* leaf epidermal cells. (a) Localization of TE‐2‐6b:RFP in the absence of TCP4:GFP. Left, visible light channel; middle, RFP channel; right, combined visible light and RFP channel. Inset: individual nucleus with nucleolus. Left, visible light channel; right, RFP channel. The nucleolus is less stained. (b) Co‐localization of TCP4:GFP and TE‐2‐6B:RFP. Different types of transformed epidermal cells are found in the same leaf area. Arrow 1: cell transformed by *35S:TE‐2‐6b:RFP* only. Localization of TE‐2‐6B:RFP in the nucleus and cytoplasm. Arrow 2: cell transformed by *35S:TCP4:GFP* only; TCP4:GFP in nuclear foci. Arrow 3: cell transformed by *35S:TCP4:GFP* and *35S:TE‐2‐6b:RFP*, co‐localization of TCP4:GFP and TE‐2‐6B:RFP in nuclear foci and cytoplasmic spots. (c) Localization of TE‐2‐6B:RFP in nuclear spots upon co‐expression with TCP4:GFP. Arrows marked 1: nuclei of cells that only express the TE‐2‐6B:RFP protein, with a homogeneous TE‐2‐6B:RFP distribution. Arrows marked 2: nuclei of cells that express both TE‐2‐6B:RFP and TCP4:GFP. TE‐2‐6B:RFP is localized in nuclear foci and coincides with TCP4:GFP localization. (d) A leaf cell in which the upper part contains cytoplasmic spots with coinciding TCP4:GFP and TE‐2‐6B:RFP signals, whereas the lower part only contains TE‐2‐6B:RFP. (e) A leaf cell in which the upper part only contains large cytoplasmic spots with coinciding TCP4:GFP/TE‐2‐6B:RFP signals, whereas the lower part contains both large and small spots with coinciding TCP4:GFP/TE‐2‐6B:RFP signals. Panels in (b)–(e) are as follows: top left, G, GFP channel; top right, RFP channel; bottom left, combined GFP and RFP channels; bottom right, visible light channel. Scale bars: 100 μm.

In order to investigate the intracellular co‐localization of TE‐6B and TCP proteins, the following *6b‐RFP* genes were constructed (see Experimental Procedures): *RFP:TE‐1‐6b‐L*, *RFP:TE‐1‐6b‐R* and *TE‐2‐6b:RFP*. These constructs were transiently expressed in *N. benthamiana* leaves. The RFP‐tagged 6B proteins were found in the cytoplasm and nucleus, with less fluorescence in the nucleolus. The localization of TE‐2‐6B:RFP is shown in Figure 7(a). The same constructs were co‐expressed with *TCP4:GFP* in *N. benthamiana* leaves. The 6B:RFP proteins co‐localized with TCP4:GFP in nuclear and cytoplasmic spots. The *TE‐2‐6b:RFP*/*TCP4:GFP* co‐infiltration results are shown in Figure 7(b)–(e). In the majority of cases, individual cells showed both GFP and RFP fluorescence. Occasionally, a mixture of cell types was observed (Figure 7b) in which some cells only expressed *TCP4:GFP* (TCP4:GFP localized in nuclear foci), others only *TE‐2‐6b:RFP* (with homogeneous TE‐2‐6b:RFP distribution) and still others both *TCP4:GFP* and *TE‐2‐6b:RFP*, with TCP4:GFP and TE‐2‐6B:RFP co‐localized in cytoplasmic spots and nuclear foci. In cells that only expressed the *TE‐2‐6b:RFP* construct (Figure 7c, arrows marked 1), TE‐2‐6B:RFP was homogeneously distributed as in the *TE‐2‐6b:RFP* control (Figure 7a). In cells expressing both *TCP4:GFP* and *TE‐2‐6b:RFP*, the TE‐2‐6B:RFP signal became partially localized in nuclear foci such as TCP4:GFP (Figure 7c, arrows marked 2), showing that co‐localization had also occurred in the nucleus. In rare cases, the distribution of the cytoplasmic spots was irregular (Figure 7d,e), suggesting some intracellular heterogeneity in cytoplasmic spot formation.

In order to test the effects of 2×*35S:TE‐2‐6b* on additional TCP proteins, *35S:G3GFP:TCP1*, *35S:G3GFP:TCP3*, *35S:mRFP:TCP8*, *35S:GFP:TCP10, 35S:G3GFP:TCP14* and *35S:G3GFP:TCP15* constructs (Table S1) were co‐infiltrated with 2×*35S:TE‐2‐6b*. *TCP1* belongs to the class II *CYC*/*TB1 TCP* gene family and *TCP3* and *TCP10* to the class II *CIN*‐like *TCP* gene family (like *TCP2* and *TCP4*), whereas *TCP8*, *TCP14* and *TCP15* belong to class I*.* Only *35S:GFP:TCP10* formed cytoplasmic spots when co‐infiltrated with 2×*35S:TE‐2‐6b* (Figure S4, overview and detail).

The striking change in localization of TCP2:GFP, TCP4:GFP and GFP:TCP10 in the presence of TE‐2‐6B and the co‐localization of TCP4:GFP with TE‐2‐6B:RFP in nuclear foci and cytoplasmic dots suggested that the TE‐6B proteins form complexes with TCP proteins. We next tested whether TE‐6B and TCP proteins interact in a direct or indirect way by performing a yeast double‐hybrid assay.

### The TCP and TE‐6B proteins interact in yeast double‐hybrid experiments

**Figure 8:**
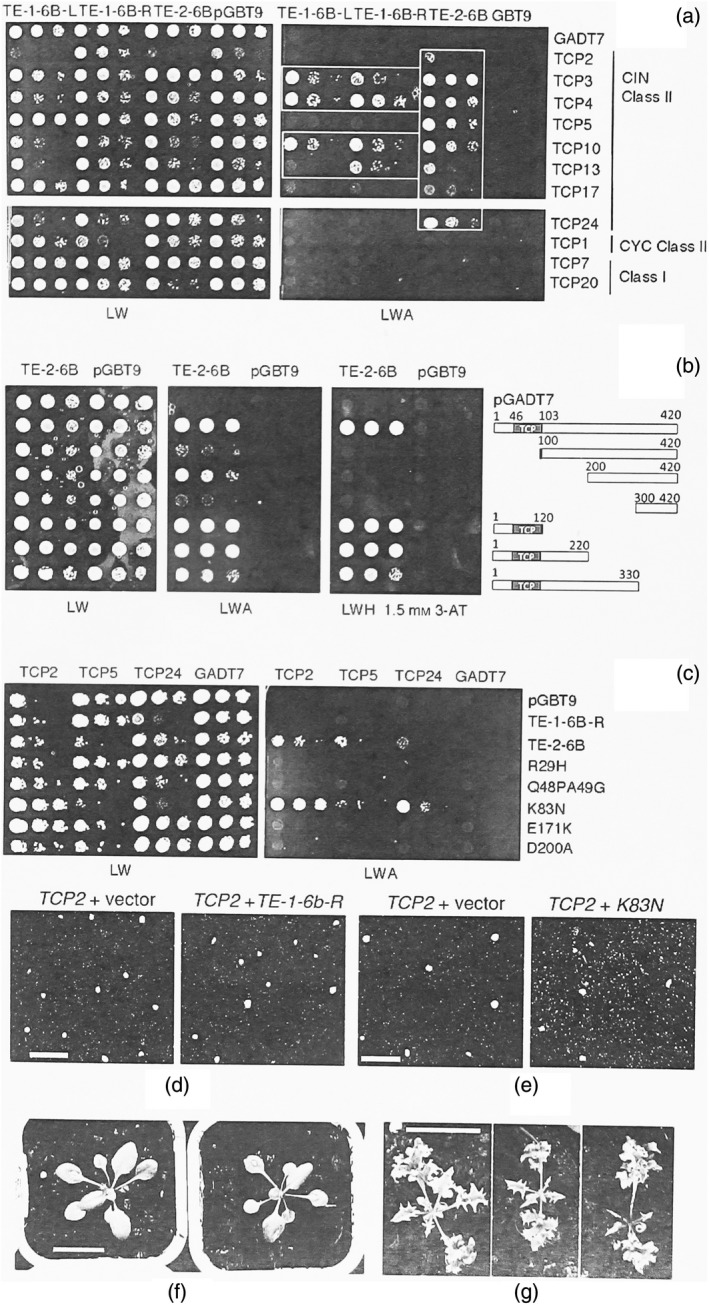
Interaction between TE‐1‐6B‐L, TE‐1‐6B‐R and TE‐2‐6B and different TCPs in a yeast double‐hybrid assay. (a) Interaction between TE‐1‐6B‐L, TE‐1‐6B‐R and TE‐2‐6B (cloned into pGBT9) and different TCP proteins (cloned into pGADT7). LW, selective LW control plates; LWA, selective LWA plates. TE‐2‐6B interacts with all members of the CIN class II TCPs, whereas the interactions of TE‐1‐6B‐L and TE‐1‐6B‐R are restricted to TCP3, ‐4 and ‐10, with TE‐1‐6B‐R additionally interacting with TCP13. No interactions are seen with the CYC class II member TCP1 or class I members TCP7 and 20. (b) Binding of TE‐2‐6B (cloned into pGBT9) to TCP4 deletion variants (cloned into pGADT7). pGBT9 and pGADT7, empty vectors; LW, non‐selective plate. On selective LWA medium and the more selective LWH + 1.5 mm 3‐amino‐1,2,4‐triazole (3‐AT) medium, the smallest TCP4 variant (N‐terminal amino acids 1–120) still shows growth. The TCP4 DNA‐binding domain (marked TCP) is shown in grey. (c) Increase in TCP‐binding capacity by change of individual TE‐1‐6B‐R residues to the corresponding TE‐2‐6B residues. Only the K83N mutation allows TE‐1‐6B‐R to bind to TCP2, ‐5 and ‐24 like TE‐2‐6B. (d) Co‐infiltration of *TCP2:GFP* along with the vector (left) or with *TE‐1‐6b‐R* (right). No change in TCP2:GFP localization. (e) Co‐infiltration of *TCP2:GFP* with the vector (left) or with the *TE‐1‐6b‐R K83N* mutant (right). The *TE‐1‐6b‐R K83N* mutant changes the TCP2:GFP localization from nuclear to nuclear and cytoplasmic. (f) *TE‐1‐6b‐R* Col‐0 plants show no obvious phenotype. (g) *TE‐1‐6b‐R K83N* Col‐0 plants are smaller and strongly crinkled. Scale bars: 200 μm (d,e); 2 cm (f,g).

Interactions between the three TE‐6B proteins and various TCP proteins (Tables S2 and S3) were investigated with a yeast double‐hybrid assay (see Experimental Procedures). The results (Figure 8a) showed that the three TE‐6B proteins can bind to TCP proteins in the absence of other plant proteins and that ‘weak’ and ‘strong’ TE‐6B proteins do not bind to the same TCP subsets despite their high sequence similarity. While TE‐1‐6B‐L interacted with TCP3, ‐4, and ‐10 and TE‐1‐6B‐R with TCP3, ‐4, ‐10 and ‐13, TE‐2‐6B bound to the full set of CIN‐like class II TCP proteins (i.e. TCP2, ‐3, ‐4, ‐5, ‐10, ‐13, ‐17 and ‐24). None of the TE‐6B proteins bound to TCP1 (CYC/TB‐type class II), TCP7 or TCP20 (class I). The interaction pattern of TE‐2‐6B with TCP4 deletion variants (Figure 8b) showed that TE‐2‐6B strongly interacted with the 120‐amino‐acid fragment that contains the 58‐amino‐acid TCP DNA‐binding domain (Aggarwal *et al.*, 2010). However, the C‐terminal 200–420 part of TCP also showed some binding interaction.

### A single nucleotide change increases the TE‐1‐6B‐R TCP‐binding repertoire and leads to a crinkly phenotype

Unlike TE‐2‐6B, TE‐1‐6B‐R did not interact with TCP2, ‐5, ‐17 or ‐24 in the yeast double‐hybrid assay, despite the fact that these two TE‐6B proteins only differ at five positions (Chen *et al.*, 2018). In order to test the importance of these differences for generating the crinkly phenotype, we replaced each of the TE‐1‐6B‐R residues with the corresponding TE‐2‐6B residue, thus producing five mutants (R29H, Q48PA49G, K83N, E171K and D200A). Each mutant was tested in the yeast double‐hybrid system by using TCP2, ‐5 and ‐24 as targets. The K83N mutant acquired the capacity to bind to TCP2, ‐5 and ‐24, whereas the other mutants did not (Figure 8c). A further test with the complete set of CIN class II TCPs showed that the K83N mutant bound all of these TCPs, similar to TE‐2‐6B (Figure S5). Thus, the asparagine residue at position 83 is essential for binding to TCP2, ‐5, ‐17 and ‐24. When the asparagine is replaced by lysine, the protein loses its ability to bind to these TCPs, although its binding interaction with TCP3, ‐4, ‐10 and ‐13 is not affected. The *TE‐1‐6b‐R* and *TE‐1‐6b‐R K83N* gene constructs were subsequently placed under 35S promoter control (see Experimental Procedures). In transient co‐expression assays in *N. benthamiana*, *TE‐1‐6b‐R* did not lead to cytoplasmic TCP2:GFP spots (Figure 8d), but the *K83N* mutant did (Figure 8e). *Arabidopsis thaliana* Col‐0 plants were stably transformed with the same constructs. Whereas the *TE‐1‐6b‐R* plants had no obvious mutant phenotype (Figure 8f), most *TE‐1‐6b‐R K83N* plants were smaller and showed crinkly leaves (Figure 8g).

### TE‐2‐6B represses the transcriptional activity of TCP4 in yeast

**Figure 9:**
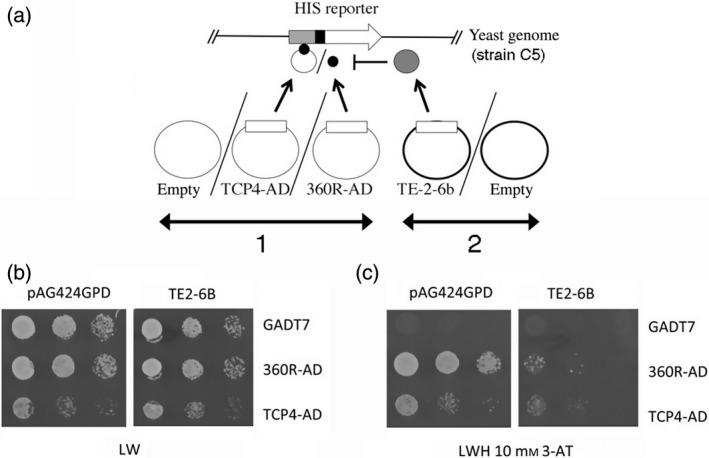
TE‐2‐6B blocks activation of an artificial target gene by TCP4 and its DNA‐binding domain in yeast. (a) Experimental setup. A HIS‐reporter gene with a minimal promoter (black) and a multimer of the TCP4 target sequence (grey) was integrated into the yeast genome, yielding strain C5. Strain C5 was transformed with a combination of two plasmids: (1) Empty vector pGADT7, pGADT7:TCP4‐AD, or pGADT7:360R‐AD and (2) Empty expression vector pAG424GPD or pAG424GPD:TE‐2‐6B. The small black disk represents the TCP4 DNA‐binding domain. (b) On non‐selective LW medium, the GADT7‐ and 360R‐AD‐carrying C5 strains grew well in the presence of the empty pAG424GPD vector (left part). TCP4‐AD grew less well, as noted earlier (Aggarwal et al., 2010). The growth observed in the presence of pAG424GPD:TE‐2‐6B (right part) was similar to that observed in the presence of pAG424GPD. (c) On the selective LWH 10 mm 3‐AT medium, the GADT7‐carrying C5 strain did not grow in the presence of the empty pAG424GPD vector (left part), while strains carrying 360R‐AD and TCP4‐AD grew well, showing activation of the HIS construct by the TCP4 DNA‐binding domain and full‐size TCP4 protein. In the presence of pAG424GPD:TE‐2‐6B (right part), the growth of the C5 strains carrying 360R‐AD and TCP4‐AD was strongly diminished, showing that TE‐2‐6B blocks transcriptional activity of the reporter gene by the TCP4 DNA‐binding domain and full‐size TCP4 protein.

Fusion proteins containing a GAL4 activation domain linked to the full‐size TCP4 protein or to the TCP4 DNA‐binding domain can activate a chromosomally located HIS‐reporter construct, placed under the control of a minimal promoter and a multimeric consensus TCP4‐binding site (Aggarwal *et al.*, 2010). We used this assay to test the capacity of TE‐2‐6B to interfere with the binding of the TCP4 protein (TCP4‐AD) or its DNA‐binding domain (360R‐AD) to the corresponding DNA target (see Experimental Procedures). First, the HIS‐reporter construct was integrated into the yeast nuclear genome, yielding strain C5. C5 was transformed with the empty vector GADT7 or with GADT7 carrying TCP4‐AD or 360R‐AD. Each of the three strains was transformed with either the empty pAG424GPD vector or the pAG424GPD:TE‐2‐6B construct. The setup of this experiment is shown in Figure 9(a). C5 strains containing pAG424GPD with either GADT7, TCP4‐AD or 360R‐AD grew well on LW control medium (Figure 9b). The strain with the TCP4‐AD construct showed lower growth than that carrying the GADT7 control. This inhibitory effect of TCP4 has been noted before (Aggarwal *et al.*, 2010). Replacement of pAG424GPD with pAG424GPD:TE‐2‐6B did not modify these results. On selective LWH medium with 10 mm 3‐amino‐1,2,4‐triazole (3‐AT) (Figure 9c), the strain with the empty GADT7 vector did not grow at all, while those with the 360R‐AD and TCP4‐AD constructs grew well, demonstrating the binding of the full‐size TCP4 and the fragment with the TCP4 DNA‐binding domain to the promoter construct and subsequent activation of transcription, as expected. However, in the presence of TE‐2‐6B, growth on the selective medium was significantly reduced, showing that the TE‐2‐6B protein not only binds to the TCP4 DNA‐binding domain but also blocks the transcriptional activation process.

## Discussion

*Agrobacterium* T‐DNA *6b* genes induce striking and highly specific phenotypes in different host plants. Various mechanisms have been proposed for their mode of action. AK‐6B binds to the nuclear proteins NtSIP1, NtSIP2 and histone H3 (Kitakura *et al.*, 2002; Terakura *et al.*, 2007; Kitakura *et al.*, 2008), which might lead to changes in transcription and subsequent growth modifications (Ishibashi *et al.*, 2014; Ito and Machida, 2015). AK‐6B and AB‐6B have been found to ADP‐ribosylate AGO1 and SERRATE proteins and modify miRNA patterns in Arabidopsis, which has been proposed to lead to the *AK‐6b*‐associated serrate phenotype (Wang *et al.*, 2011). Another study had found that 6B proteins stimulate the local uptake and retention of sucrose in both leaves (Clément *et al.*, 2006) and roots (Clément *et al.*, 2007), a process which might induce the observed ectopic primordia (Chen and Otten, 2016); however, the molecular basis for 6B‐enhanced sucrose uptake remains unknown. The present study demonstrates an unexpected link between the TE‐6B proteins encoded by the TE cT‐DNAs from the natural transformant *N. otophora* and the well‐known TCP transcription factors. First of all, a striking resemblance was noted between *TE‐2‐6b *Arabidopsis plants and Arabidopsis lines with decreased class II CIN‐like TCPs levels (like *jaw‐D* mutants). Second, the three TE‐6B proteins were found to associate with several CIN‐like TCP proteins both *in planta* and in yeast. The similarities between the phenotypes and transcriptional patterns of *TE‐2‐6b* plants and the *jaw‐2D* mutant provide strong evidence for the biological relevance of these molecular interactions. It has been proposed that downstream targets of CIN‐like TCPs control cell division arrest at leaf margins in the early stages of leaf development (Nath *et al.*, 2003; Bresso *et al.*, 2018). According to this model, decreased TCP levels lead to an abnormal continuation of cell division at the leaf edge, causing crinkly leaves. The increase in surface area at the edge of the leaf, combined with the physical forces operating in thin sheets, is most likely sufficient to explain the fractal crinkling patterns (Sharon *et al.*, 2002) typical of *jaw‐D*, *SAP11* and *TE‐2‐6b* plants. The leaf edges of Col‐0 *TE‐2‐6b* plants with a strong phenotype also form small finger‐like structures. These may be related to the tubular structures growing from the abaxial side of *AB‐6b *Arabidopsis leaves (Helfer *et al.*, 2003) or behind the glandular trichomes of *T‐6b N. tabacum* plants. The latter can develop into small ectopic leaves, which shows that they are abnormal leaf primordia (Chen and Otten, 2016). The origin, development and underlying cell division patterns of the tubular Col‐0 *TE‐2‐6b* leaf structures merit further analysis.

Co‐expression of the TE‐6B proteins with TCP2, ‐4 or ‐10 in *N. benthamiana* led to remarkable changes in the cellular localization of both TE‐6B and TCP proteins. While TCP2, ‐4 and ‐10 are normally found in nuclear foci, they partially relocalized to numerous cytoplasmic spots in the presence of TE‐2‐6B. Conversely, TE‐2‐6B, when expressed alone, was homogeneously distributed in the cytoplasm and nucleus (with the exception of the nucleolus); however, in the presence of TCP2, ‐4 or ‐10 it became co‐localized with the TCPs in cytoplasmic spots and nuclear foci. In *N. benthamiana,* TCP3:GFP localization was not influenced by TE‐2‐6B, although binding did occur in yeast. The reason for this discrepancy is unknown. The effects of TE‐6B proteins on the localization of the other CIN‐like TCPs (TCP5, ‐13, ‐17 and ‐24) remain to be studied.

It is interesting to note that while *SAP11* expression caused loss of TCP proteins, *TE‐2‐6b* expression increased the levels of TCP2 and TCP4. This remarkable difference may be due to the fact that SAP11 is localized in the nucleus (Sugio *et al.*, 2014), where TCP degradation takes place (Mazur *et al.*, 2017). TE‐6B proteins, on the contrary, are present in the nucleus and cytoplasm. They lack nuclear import or export signals – unlike SAP11 (Sugio *et al.*, 2014) – and can bind CIN‐TCP proteins in the cytoplasm. TE‐6B proteins could exert their effects in two ways: by retaining CIN‐TCPs in the cytoplasm and by interfering with CIN‐TCP transcriptional activation in the nucleus. The relative importance of these two activities will depend on the binding properties, accumulation kinetics and stability of the 6B/TCP complexes in both compartments. Further experiments are required to measure these parameters. Addition of the strong universal SV40 nuclear localization signal of the yeast pGADT7 vector to TCP4 does not prevent the inhibitory effect of the *TE‐2‐6b* gene, showing that cytoplasmic retention is not essential.

We postulate that TE‐6B/TCP binding in the cytoplasm prevents or slows down degradation of TCP, although this remains to be demonstrated. In yeast, TE‐2‐6B, TE‐1‐6B‐L and TE‐1‐6B‐R were found to bind different subsets of TCP proteins. Differences in TCP‐binding patterns were also noted for some SAP11 variants (Chang *et al.*, 2018; Wang *et al.*, 2018). Strong Arabidopsis TCP phenotypes require simultaneous inactivation of several *TCP* genes (Efroni *et al.*, 2008; Schommer *et al.*, 2008; Koyama *et al.*, 2010; Alvarez *et al.*, 2016; Bresso *et al.*, 2018). We propose that the differences between the ‘weak’ and ‘strong’ TE‐6b phenotypes are caused by differences in TCP‐binding properties. We have shown that a single amino acid change (K83N) in the ‘weak’ TE‐1‐6B‐R protein extends its TCP‐binding repertoire in yeast to that of the ‘strong’ TE‐2‐6B protein and generates a crinkly phenotype in Arabidopsis. Out of 21 6B proteins, 15 carry asparagine, 4 serine and 2 (TE‐1‐6B‐L and TE‐1‐6B‐R) lysine at position 83 (Chen *et al.*, 2018). In addition, the Plast proteins that are most closely related to 6B (6A, Orf14 and RolC) also carry asparagine at the corresponding position (Helfer *et al.*, 2002). Thus, TE‐6B proteins with lysine at position 83 seem to be derived from those with an asparagine residue.

*TE‐2‐6b* expression caused a strong decrease in the mRNA levels of the TCP4 target gene *LOX2* in Arabidopsis. In yeast, TE‐2‐6B directly targeted the TCP4 DNA‐binding domain and blocked TCP4‐dependent expression of a reporter gene carrying the TCP4‐binding site. This experimental system could be useful for further TE‐6B/TCP interaction studies and for identifying TE‐6B‐resistant TCP variants.

It has been noted that different plant pathogens target TCPs (Danisman, 2016; Dhaka *et al.*, 2017). Apart from Aster Yellows phytoplasma, which employs the SAP11 effector, *Pseudomonas syringae* uses the HopBB1 effector to interact with the class I TCP14 protein and present it to the SCF‐degradation complex, thus de‐repressing TCP14‐controlled jasmonate response genes (Yang *et al.*, 2017). Our results show that the plant pathogen *Agrobacterium* can also target TCP proteins by using a protein unrelated to SAP11.

Although the TE‐6B/TCP interactions might be sufficient to explain the *jaw*‐like phenotype of *TE‐2‐6b* plants, it cannot be excluded that the TE‐6B proteins also target other proteins, which might include some of those reported in studies with other 6B proteins. Conversely, these other 6B proteins might also bind TCP proteins. In Arabidopsis, *AK‐6b* induces a serrate phenotype (Terakura *et al.*, 2006) similar to the weaker *TE‐2‐6b* phenotypes. However, *AK‐6b* plants lack the strong outgrowth and wrinkling of leaf edges and petal and silique modifications typical of *TE‐2‐6b* plants. In view of the large sequence differences between TE‐6B proteins and the oncogenic 6B proteins like AB‐6B, it is possible that the latter do not bind to TCP proteins. However, if they do, TCP proteins may play a role in the arrest of wound‐induced cell divisions. Inactivation of TCPs by oncogenic 6B proteins could then lead to prolonged cell division at the wound site. Further studies are required to test this possibility.

The expression patterns of *TE‐6b* and *TCP* genes in *N. otophora* are largely unknown, and it is important to study whether and how they overlap. However, it should be noted that *TE‐2‐6b*‐induced changes in vein growth are graft‐transmissible (Chen *et al.*, 2018). Thus, *TE‐2‐6b* could affect TCP activities beyond its domain of expression. Different 6B proteins may produce mobile factors (enation and venation factors) with differences in mobility, stability and target‐tissue specificity, all of which could contribute to the creation of different *6b* phenotypes. Further research is needed to identify these mobile factors, their target tissues, and possible interactions with TCP proteins at the target sites.

Our results show that the transfer of a partial, inverted T‐DNA repeat from *Agrobacterium* to an *N. otophora* ancestor, its subsequent duplication to TE‐1 and TE‐2, and the loss of the right‐hand part of the TE‐2 repeat were accompanied by structural and functional divergence of the *TE‐6b* genes. The effects of these changes on the growth of *N. otophora* may be further investigated by removal of the resident *TE‐6b* genes and introduction of *6b* gene variants. Finally, the present results are expected to be of benefit to both the TCP and 6B research areas and might lead to new tools for improving plant growth.

## Experimental Procedures

### Transformation of Arabidopsis Col‐0

*Arabidopsis thaliana* ecotype Col‐0 plants were transformed by floral dip as described by Helfer *et al.* (2003) and allowed to set seed. Seeds were selected on 0.5× Murashige and Skoog medium with 50 mg L^−1^ kanamycin and 350 mg L^−1^ Claforan. Lines with a single T‐DNA locus were selected by segregation analysis of F_2_ seeds on kanamycin, and homozygous lines were identified in the F_3_ generation by selection on kanamycin.

### *jaw‐D* mutants

*jaw‐D* mutants *jaw‐1D* (N6948) and *jaw‐2D* (N6949) were obtained from the Nottingham Arabidopsis Stock Centre collection (http://arabidopsis.info/).

### Transient expression in *Nicotiana benthamiana*

Transient expression was achieved by agroinfiltration of *N. benthamiana* leaves (Yang *et al.*, 2000). Overnight bacterial cultures were washed with one volume 10 mm 2‐(*N*‐morpholino)ethanesulfonic acid pH 5.8, 5 mm MgCl_2_ and 0.15 mm acetosyringone and resuspended in the same buffer to an optical density of 0.4 at 600 nm. A *P19* silencing suppressor gene construct (Voinnet *et al.*, 1999) was added to increase expression. Mixtures were prepared in a ratio of 1:1:1 (v/v/v) as required. LBA4404(pBI121.1) (Jefferson *et al.*, 1987) was used as the empty vector control. Observations were made 48 h after infiltration.

### Microscopy

Laser scanning confocal microscopy of plant tissue was performed by using a Zeiss LSM780 microscope with a Plan‐Apochromat 20×, 0.8 NA objective lens. Green fluorescent protein, chlorophyll and monomeric RFP (mRFP) were excited using the 488 nm argon and 561 nm diode‐pumped solid‐state laser lines. For optimal single‐track multichannel dye separation, the GFP signal was detected using Band Pass filter (BP) 493–556 nm, and mRFP fluorescence using BP 606–641 nm, minimizing spectral overlap. The chlorophyll channel was collected using BP 684–749 nm. Transmitted light images were simultaneously collected in the transmission photomultiplier tube channel. Low‐resolution fluorescence microscopy was performed with a Zeiss Axiozoom V16 Apotome.2 microscope.

### Yeast double‐hybrid assays and yeast constructs

Growth, plating and selection were performed by using the Yeast Protocols Handbook (Takara, https://www.takara-bio.com) and Matchmaker Gal4 Two‐Hybrid System 3 and Libraries User manual (Clontech). Yeast transformation was performed as described by Gietz and Schiestl (2007). The yeast strain PJ69‐α was used for the yeast double‐hybrid assay (James *et al.*, 1996). Yeast one‐hybrid assay strain creation and testing were performed following the protocols of Fuxman Bass *et al.* (2016), with the exception that yeast strain INCSc1 (ThermoFisher Scientific, https://www.thermofisher.com/) was used in this study. Bait constructs were made by LR recombination in a Gateway‐converted pGBT9 vector (Clontech). AD constructs were made by standard cloning in pGADT7 (Clontech), using various TCP primers (Table S3). pGADT7‐TCP3, pGADT7‐TCP4 and pGADT7‐TCP10 are described by Li *et al.* (2012). The *TCP5* open reading frame was amplified from genomic DNA. Coding sequences for *TCP1*, *TCP2*, *TCP7*, *TCP13*, *TCP17*, *TCP20* and *TCP24* were amplified from pGADT7 Gateway destination vector constructs (Davière *et al.*, 2014). *TCP* coding sequences were ligated to a *Bam*HI–*Xho*I‐digested pGADT7 or pENTR1A vector (Clontech). Similarly, deletion constructs of *TCP4* were prepared in a *Bam*HI–*Xho*I‐digested pGADT7 vector. For the one‐hybrid experiments, a double‐stranded gBlocks oligonucleotide containing 12 copies of the TCP4 binding sequence GTGGTCCC and one 3′ *Sma*I restriction site was cloned into the pGemT Easy vector (Promega, https://www.promega.com/). Several positive clones were isolated and found to have 24 copies of the TCP‐binding sites and a deleted internal *Sma*I restriction site, but an intact 3′ *Sma*I site. The resulting 24‐copy TCP4‐binding site was excised by *Eco*RI and *Sma*I digestion and ligated to an *EcoR*I–*Sma*I‐digested pHISi‐1 vector (Clontech). *TE‐2‐6b* was transferred to the pAG424PDG‐ccdB vector (Addgene, https://www.addgene.org/) by LR recombination. Site‐directed mutagenesis of *TE‐1‐6b‐R* was performed in a pENTR3C clone using the Quikchange (Agilent, https://www.agilent.com/) protocol. All constructs were verified by DNA sequencing.

### RNA gel‐blot analysis 

Total RNA was extracted from Arabidopsis leaves 5 weeks after sowing using TRI Reagent (Sigma, https://www.sigmaaldrich.com/) in accordance with the manufacturer's instructions. Low‐molecular‐weight RNA gel‐blot analysis was performed with 20 μg of total RNA as described previously (Montavon *et al.*, 2017). The DNA probes were radiolabelled with [γ‐^32^P]ATP by using T4 PNK (Thermo Scientific, https://www.thermofisher.com/): miR319a probe 5′‐GGGAGCCTCCCTTCAGTCCAA‐3′; U6 loading control probe 5′‐AGGGGCCATGCTAATCTTCTC‐3′. Hybridization was performed overnight in PerfectHyb Plus (Sigma‐Aldrich, St. Quentin Fallavier Cedex, France) at 42°C and the membranes were washed three times in 2% sodium dodecyl sulfate and 2× saline–sodium citrate buffer at 50°C.

### Quantitative PCR (qPCR) assay

Samples for RNA extraction were prepared as pools of RNA (in triplicate) from five 10‐day‐old seedlings of each genotype: Col‐0, *jaw‐2D* and *TE‐2‐6b* line 21‐2. Reverse transcription was performed using 0.5 μg of RNA. Quantitative PCR was performed for the genes *TCP4*, *LOX2* and *TE‐2‐6b* using the following primers: 5′‐GAGCTCTCTTGTTTATCACCTGCGTTTATAAACAAG‐3′/5′‐AGCAACCGATACAGGAAACGGA‐3′ (*TCP4*), 5′‐AGGACTCATGCCTGTACGGAGCCA‐3′/ and 5′‐AACTCCGCCAACTTTGGAT‐3′/5′‐GCTCCCGAAGAGCTTGATT‐3′ (*TE‐2‐6b*). The expression levels of *PROTEIN PHOSPHATASE 2A* mRNA (*PP2A*) were used for normalization. Statistical significance was determined by the unpaired two‐sample Student's *t*‐test by using Infostat software. *P*‐values are indicated in the figure legends.

### Transcriptome analysis

Plant material was collected 5 weeks after sowing. Total RNA was extracted from leaves by using TRI Reagent (Sigma) in accordance with the manufacturer's instructions. Libraries were generated using an Illumina TruSeq Stranded Kit (Illumina Inc., https://www.illumina.com/). Normalized libraries were pooled and clustered on an Illumina HiSeq 3000/4000 PE flow cell by using an Illumina cBot system and an Illumina HiSeq 3000/4000 PE Cluster Kit. The flow cell was sequenced on an Illumina HiSeq 4000 using the Illumina HiSeq 3000/4000 SBS Kit (300 cycles) in a 2 × 151 paired‐end mode. The reads were demultiplexed, and fastp was used to remove Illumina adapters, discard low‐complexity reads and trim low‐quality trailing bases. Only paired reads with a minimum length of 75 bp were retained. The reads were pseudoaligned to the Arabidopsis transcriptome and *TE‐2‐6b* transcript sequence using kallisto. Transcript‐level estimated read counts were aggregated to gene level using the tximport R package, and gene expression was analyzed with the DESeq2 R package. Principal component analysis was performed with the PCAtools R package using the 5% of genes with the highest variability. The Illumina sequencing data will be made available upon request.

### Gene constructs for plant expression

The 2×*35S:TE‐1‐6b‐L*, 2×*35S:TE‐1‐6b‐R* and 2×*35S:TE‐2‐6b* constructs have been described previously (Chen *et al.*, 2018). *GFP* and *RFP* fusion genes were constructed in two steps. *TE‐6b* genes were amplified from *N. otophora* genomic DNA. After addition of *Bam*HI and *Xho*I sites, the genes were cloned into a *Bam*HI–*Xho*I‐digested pENTR3C vector. *TCP10* was introduced into a pENTR3C vector as an *Eco*RI–*Xho*I fragment from pGADT7‐TCP10. After sequencing, the genes were introduced into pK7FWG2 (for a C‐terminal GFP tag), pK7WGF2 (for an N‐terminal GFP tag), pH7RWG2 (for a C‐terminal RFP tag) and pH7WGR2 (for an N‐terminal RFP tag) destination vectors (Karimi *et al.*, 2005) by LR recombination and the constructs were checked by digestion with different restriction enzymes. The *TE‐1‐6b‐R* and *TE‐1‐6b‐R K83N* mutant genes were introduced from pENTR3C into pB7WG2 (Karimi *et al.*, 2005). Binary vectors with different gene constructs were introduced in the disarmed *Agrobacterium tumefaciens* strain LBA4404 helper strain LBA4404 (Hoekema *et al.*, 1983) for transient transformation of *N. benthamiana* leaves and into GV3101(pMP90) (Koncz and Schell (1986) for floral dip transformation of *A. thaliana*.

## Author Contributions

TP and PG provided Arabidopsis transformants and carried out yeast experiments. JP and CS did the qPCR experiments. NS and NI provided the transcriptome data. YK did the Northern blot analysis. J‐MD prepared TCP constructs. LO did Western analysis, prepared RFP constructs, did the microscopy work and wrote the manuscript.

## Conflict of Interest

The authors declare no conflict of interest. N. S. and N. V. I. are employees of Philip Morris International.

## Supporting information


**Figure S1.** Heatmap of differences in expression of genes whose expression is most and least correlated with that of *TE‐2‐6b*.

**Figure S2.** Heatmap of differences in expression of 100 genes whose expression varies the most among Col‐0, *jaw‐2D* and Col‐0 *TE‐2‐6b* line 48‐4.

**Figure S3.** Heatmap of differences in expression of *CIN‐TCP* genes, and of genes whose expression is modified in *jaw‐D* lines (Schommer *et al*., 2008) or regulated by CIN‐TCP proteins (Sarvepalli and Nath, 2018).

**Figure S4.** Effect of TE‐2‐6B on localization of additional TCP proteins in *Nicotiana benthamiana*.

**Figure S5.** Binding of the TE‐1‐6B‐R K83N mutant protein to the CIN‐TCPs in yeast.

**Table S1.** TCP constructs for expression in plants.

**Table S2.** TCP constructs for expression in yeast.

**Table S3.** List of primers for the cloning of yeast constructs.

## Data Availability

Illumina sequencing data and other data will be made available upon request.
