# Supplementary figures and images for: Inhibition of Arabidopsis thaliana CIN‐like TCP transcription factors by Agrobacterium T‐DNA‐encoded 6B proteins
Source: Plant J. 2019 Dec 5;101(6):1303–17. doi: 10.1111/tpj.14591 (PMC7187390; doi:10.1111/tpj.14591)

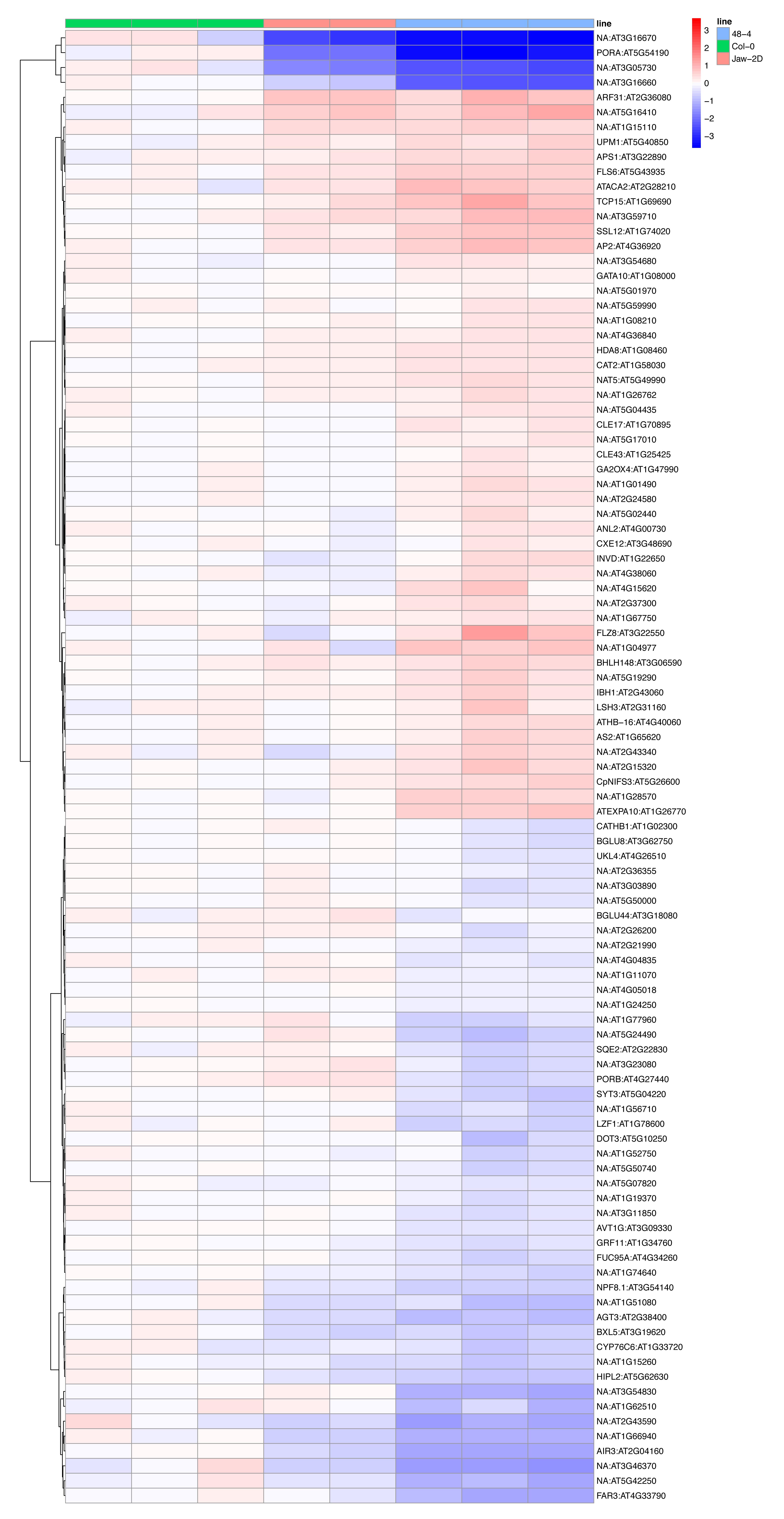

Supplement: Supplementary file 1 — Figure S1. Heatmap of differences in expression of genes whose expression is most and least correlated with that of TE‐2‐6b. [file TPJ-101-1303-s001.jpg]

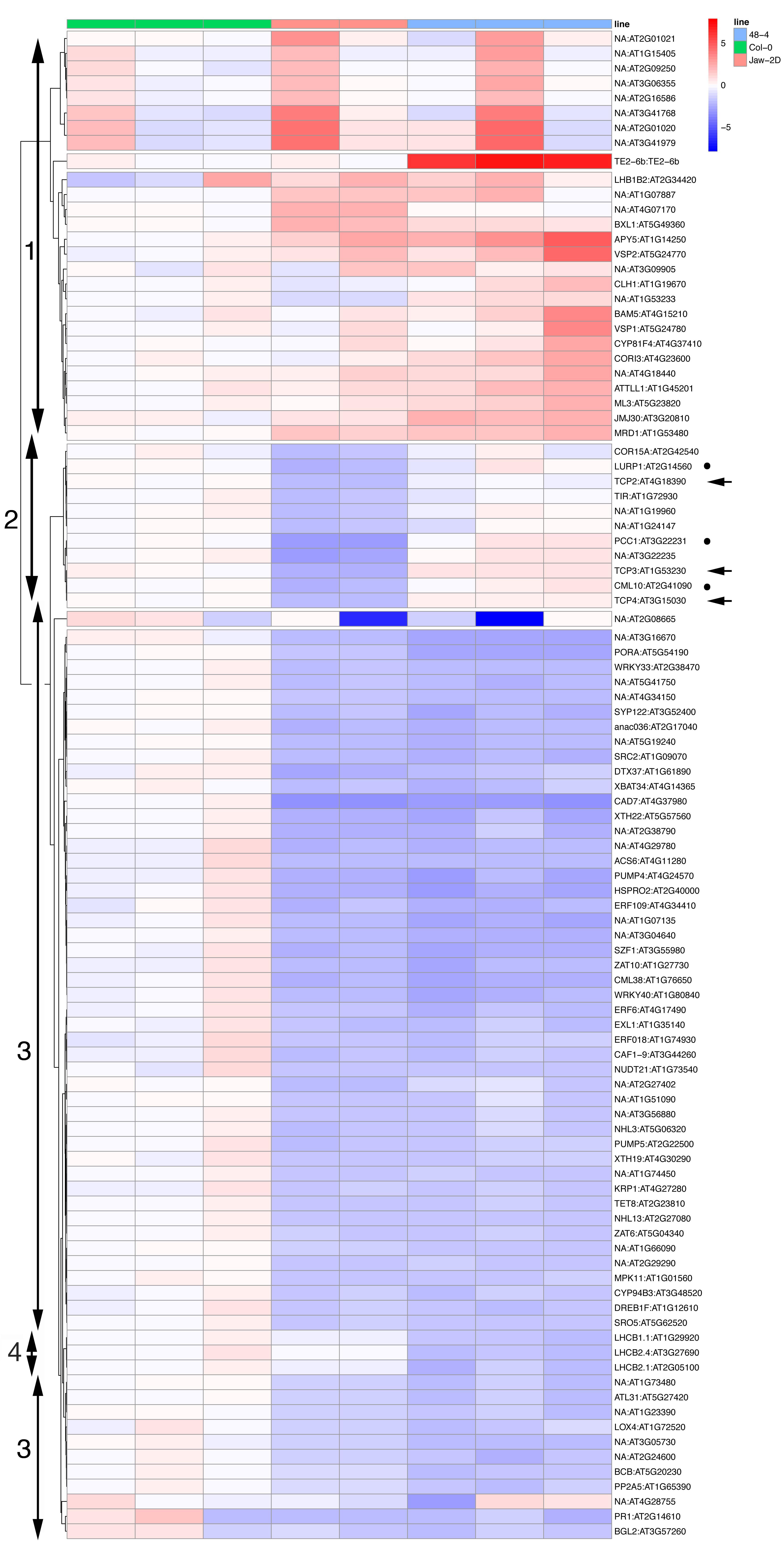

Supplement: Supplementary file 2 — Figure S2. Heatmap of differences in expression of 100 genes whose expression varies the most among Col‐0, jaw‐2D and Col‐0 TE‐2‐6b line 48‐4. [file TPJ-101-1303-s002.jpg]

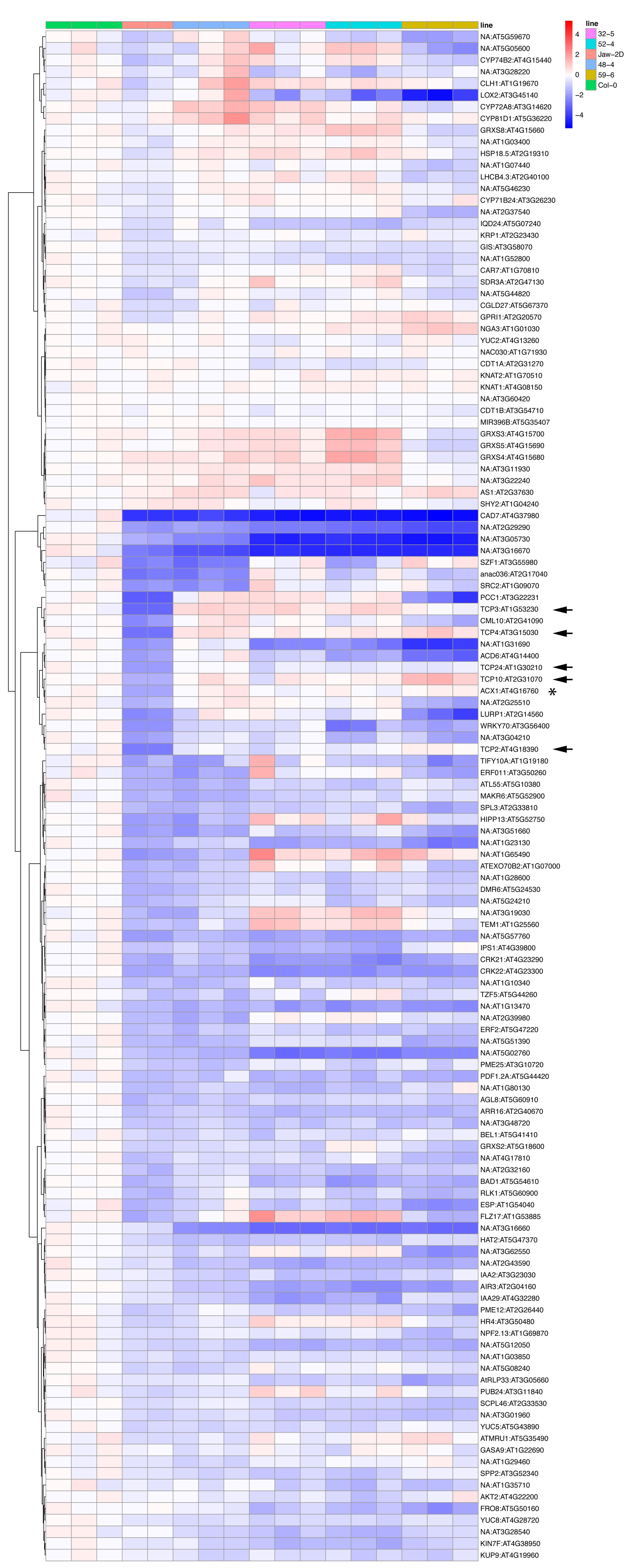

Supplement: Supplementary file 3 — Figure S3. Heatmap of differences in expression of CIN‐TCP genes, and of genes whose expression is modified in jaw‐D lines (Schommer et al., 2008) or regulated by CIN‐TCP proteins (Sarvepalli and Nath, 2018). [file TPJ-101-1303-s003.jpg]

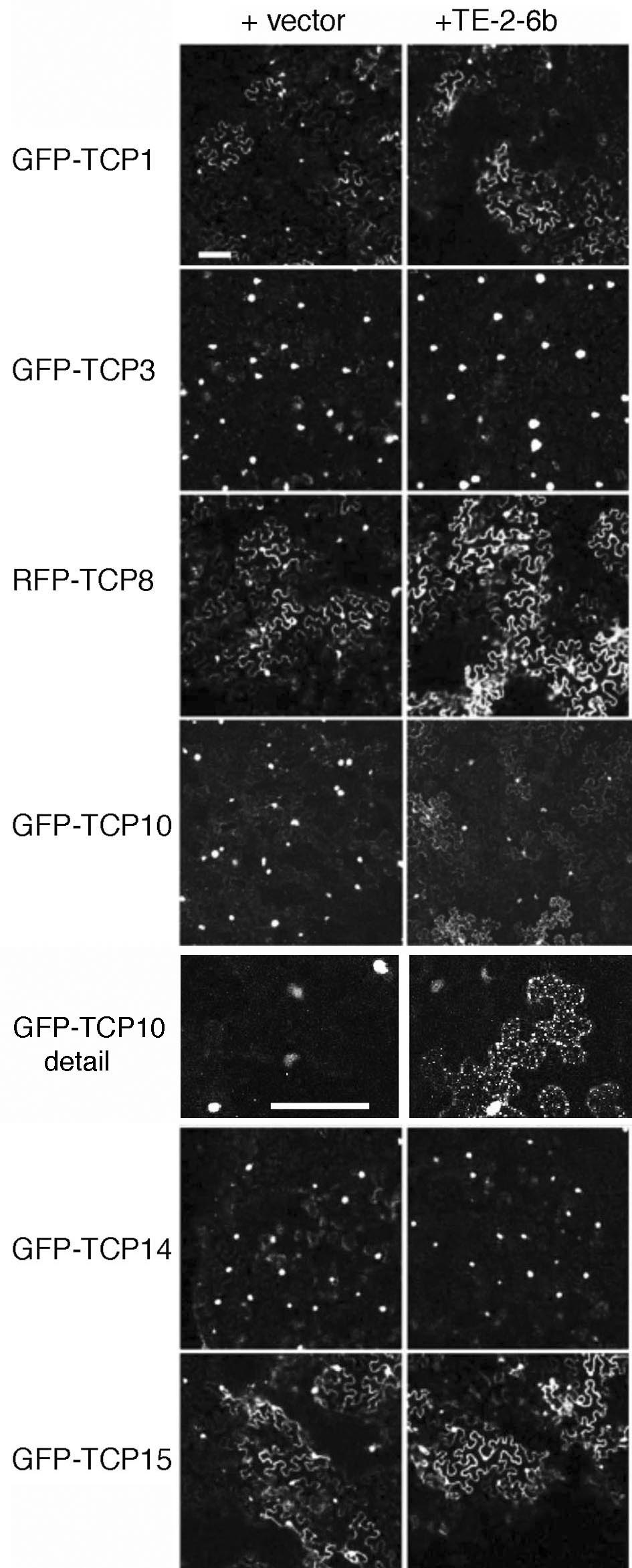

Supplement: Supplementary file 4 — Figure S4. Effect of TE‐2‐6B on localization of additional TCP proteins in Nicotiana benthamiana. [file TPJ-101-1303-s004.jpg]

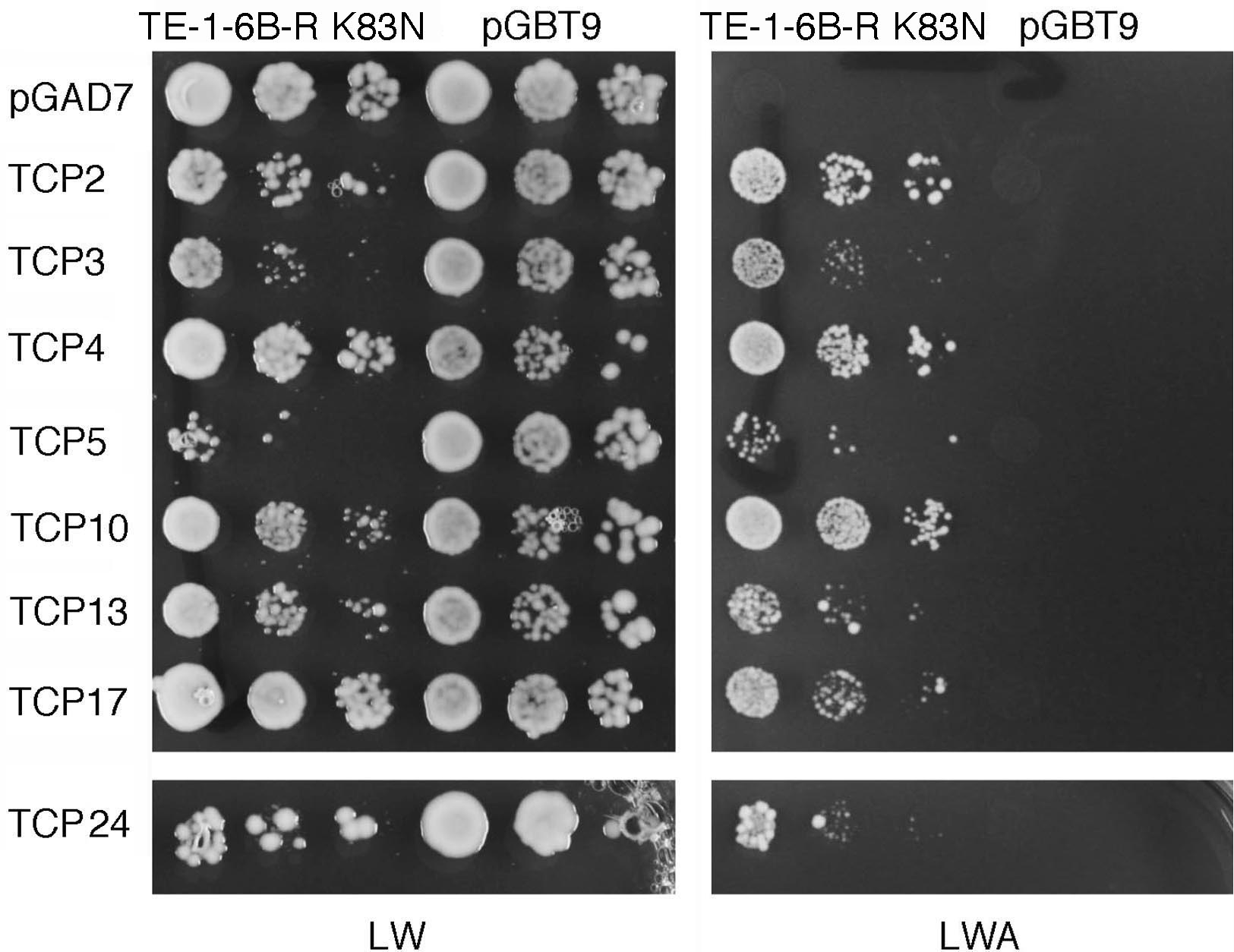

Supplement: Supplementary file 5 — Figure S5. Binding of the TE‐1‐6B‐R K83N mutant protein to the CIN‐TCPs in yeast. [file TPJ-101-1303-s005.jpg]
