# Supplementary material for: Inhibition of Arabidopsis thaliana CIN‐like TCP transcription factors by Agrobacterium T‐DNA‐encoded 6B proteins
Source: Plant J. 2019 Dec 5;101(6):1303–17. doi: 10.1111/tpj.14591 (PMC7187390; doi:10.1111/tpj.14591)
Supplement: Supplementary file 6 — Table S1. TCP constructs for expression in plants. [file TPJ-101-1303-s006.docx]

Table S1. TCP constructs for expression in plants.

| Construct | origin | Cytoplasmic dots  in presence of TE-2-6B |
| --- | --- | --- |
| GFP:TCP1 | Mazur *et al*., 2017 | - |
| TCP2:GFP | Palatnik *et al*., 2007 | + |
| GFP:TCP3 | Mazur *et al*., 2017 | - |
| TCP4:GFP | Palatnik *et al*., 2007 | + |
| RFP:TCP8 | Mazur *et al*., 2017 | - |
| GFP:TCP10 | this work | + |
| GFP:TCP14 | Mazur *et al*., 2017 | - |
| GFP:TCP15 | Mazur *et al*., 2017 | - |
