# Supplementary material for: Inhibition of Arabidopsis thaliana CIN‐like TCP transcription factors by Agrobacterium T‐DNA‐encoded 6B proteins
Source: Plant J. 2019 Dec 5;101(6):1303–17. doi: 10.1111/tpj.14591 (PMC7187390; doi:10.1111/tpj.14591)
Supplement: Supplementary file 7 — Table S2. TCP constructs for expression in yeast. [file TPJ-101-1303-s007.docx]

Table S2. TCP constructs for expression in yeast.

| construct | origin |
| --- | --- |
| pGADT7-TCP1 | this work |
| pGADT7-TCP2 | this work |
| pGADT7-TCP3 | Li *et al.,* 2012 |
| pGADT7-TCP4 | Li *et al.,* 2012 |
| pGADT7-TCP5 | this work |
| pGADT7-TCP7 | this work |
| pGADT7-TCP10 | Li *et al.,* 2012 |
| pGADT7-TCP13 | this work |
| pGADT7-TCP17 | this work |
| pGADT7-TCP20 | this work |
| pGADT7-TCP24 | this work |
