# Supplementary material for: Inhibition of Arabidopsis thaliana CIN‐like TCP transcription factors by Agrobacterium T‐DNA‐encoded 6B proteins
Source: Plant J. 2019 Dec 5;101(6):1303–17. doi: 10.1111/tpj.14591 (PMC7187390; doi:10.1111/tpj.14591)
Supplement: Supplementary file 8 — Table S3. List of primers for the cloning of yeast constructs. [file TPJ-101-1303-s008.docx]

Table S3. List of primers for cloning yeast constructs. In general, restriction sites *Bam*HI and *Xho*I were used for subcloning. Exceptions are TCP13F (*Bsa*I), TCP15F (*Bsa*I) and TCP20BsmBIF (*Bsm*BI), where the indicated Goldengate restriction sites were used to clone into a *Bam*HI-*Xho*I digested vector. The geneblocks fragment for the TCP4 binding site contains a single *Sma*I restriction site.

| name of primer | Primer sequence |
| --- | --- |
| TCP1F | ccggatccagatgtcgtcttccaccaatgac |
| TCP1R | ggctcgagttagtttacaaaagagtcttg |
| TCP2F | ccggatccagatgattggagatctaatgaag |
| TCP2R | ggctcgagtcagttcttgcctttaccctt |
| 612TCP5-F | ccggatccatatgagatcaggagaatgtgat |
| 612TCP5-R | ggctcgagtcaagaatctgattcattat |
| 612TCP7F | ccggatccatatgtctattaacaacaacaac |
| 612TCP7R | ggctcgagttaacgtggatcttcctctct |
| TCP8F | ccggatccagatggatctctccgacatccga |
| TCP8R | ggctcgagtcactcagagctatttgagttct |
| TCP13R | ggctcgagtcacatatggtgatcacttcc |
| 612TCP13-FBsaI | ccggtctcggatccagatgaatatcgtctcttggaaa |
| 612TCP13noSTOP | ggctcgagccagctccacctccacctccgcccatatggtgatcacttcctct |
| TCP15BsaIF | ccggtctcggatccagatggatccggatccggatcat |
| TCP15R | cctcgagctaggaatgatgactggtgct |
| TCP17F | ccggatccagatgggaataaaaaaagaagat |
| TCP17R | ggctcgagctactcgatatggtctggttgt |
| TCP20BsmBIF | cccgtctcggatccagatggatcccaagaacctaaat |
| TCP20R | ggctcgagttaacgacctgagccttgaga |
| TCP24F | ccggatccagatggaggttgacgaagacatt |
| TCP24R | ggctcgagctatctcctttcctttgccttg |
| TCP4-BDS | (gtggtccc) x 12 ggg |
| 612TCP4-Start | ccggatccatatgtctgacgaccaattc |
| 612TCP4-301F | ccggatccatgacgagctcgctgagctt |
| 612TCP4-601F | ccggatccatcataaccttatgcacaac |
| 612TCP4-901F | ccggatccatggaaacggaggagggttt |
| 612TCP4-STOP | ggctcgagtcaatggcgagaaataga |
| 612TCP4-960R | ggctcgagtcatggctgaaacgacgtcgt |
| 612TCP4-660R | ggctcgagtcaattagttcgagaaagcaaatc |
| 612TCP4-360R | ggctcgagtcaagcagcggctaggcgaattgc |
| TE6bENTRYF | cccgcgggatccatggctgttcccgcgtggcaggtccg |
| TE6bENTRYnostopR | ggcggctcgaggcaagccacaatcggcacgatcc |
| TE6bENTRYstopR | ggcggctcgagctaaagccacaatcggcacgatc |
| 612TPg86a-R | gattgcggaaatcgtgtttcgccgctcgta |
| 612TPg86a-F | tacgagcggcgaaacacgatttccgcaatc |
| 612TPa143cc146g | gcattcaccgttgaaacctggctgcgtttgggatgg |
| 612TPa143cc146gR | ccatcccaaacgcagccaggtttcaacggtgaatgc |
| 612TPg249t | aagttggcggaattggacggcaaacagcgattg |
| 612TPg249tR | caatcgctgtttgccgtccaattccgccaactt |
| 612TPg511a | ttggtcctcttcctctttctcttcctcttcttcct |
| 612TPg511aR | aggaagaagaggaagagaaagaggaagaggaccaa |
| 612TPa599c | tcggatagttcacgtaggccccgatttggtattct |
| 612TPa599cR | agaataccaaatcggggcctacgtgaactatccga |
