# Supplementary material for: Inhibition of Arabidopsis thaliana CIN‐like TCP transcription factors by Agrobacterium T‐DNA‐encoded 6B proteins
Source: Plant J. 2019 Dec 5;101(6):1303–17. doi: 10.1111/tpj.14591 (PMC7187390; doi:10.1111/tpj.14591)
Supplement: Supplementary file 9 [file TPJ-101-1303-s009.docx]

Figure S1. Heatmap of differences in expression of genes whose expression is most and least correlated with that of *TE-2-6b*. Differences in log2-transformed normalized and variance stabilized counts are reported, with red indicating an increase and blue indicating a decrease in gene expression compared with the mean Col-0 gene expression. The 50 most and 50 least correlated genes reported were selected on the basis of their Pearson correlation coefficient.

Figure S2. Heatmap of differences in expression of 100 genes whose expression varies the most among Col-0, *jaw-2D,* and Col-0 *TE-2-6b* line 48-4. Differences in log2-transformed normalized and variance-stabilized counts are reported, with red indicating an increase and blue indicating a decrease in gene expression compared with the mean Col-0 gene expression. Arrows: *CIN-TCP* genes. Dots: genes known to be regulated by *CIN-TCP* genes, downregulated in the *jaw-2D* line but not (or less regulated) in the *TE-2-6b* 48-4 line. 1, 2, 3, and 4 denote different groups of genes. 1: Expression upregulated or variable in *jaw-2D* and 48-4; 2: Expression downregulated in *jaw-2D*, but not in 48-4; 3: Expression downregulated in *jaw-2D* and in 48-4; 4 : Expression downregulated in 48-4, but not in *jaw-2D*.

Figure S3. Heatmap of differences in expression of *CIN-TCP* genes, and of those genes whose expression is modified in *jaw-D* lines (Schommer *et al*., 2018) or regulated by CIN-TCP proteins (Sarvepalli and Nath, 2018). Comparison between Col-0, *jaw-2D*, and Col-0 *TE-2-6b* lines 48-4, 32-5, 52-4, and 59-6. The transcript levels of the CIN-TCP target gene *ACX1* (marked by an asterisk) and the *CIN-TCP* genes *TCP2*, *TCP3*, *TCP4*, *TCP10*, and *TCP24* (marked by arrows) are reduced in the *jaw-2D* line but not affected in *TE-2-6b* lines, even in those with high *TE-2-6b* expression.

Figure S4. Effects of *2x35S-TE-2-6b* expression on localization of various additional TCP proteins. Only GFP-TCP10 forms cytoplasmic spots in the presence of TE-2-6B. Scale: 100 μm.

Figure S5. Binding of the TE-1-6B-R K83N mutant to the full set of CIN-like class II TCPs in yeast. The TE-1-6B-R K83N mutant has acquired the capacity to bind all class II CIN-TCPs, like TE-2-6B. pGADT7 and pGBT9: empty vectors. LW: non-selective plate. LWA: selective medium.

Table S1. TCP constructs for expression in plants.

Table S2. TCP constructs for expression in yeast.

Table S3. List of primers for cloning yeast constructs.
